# Recent advances in molecular mechanisms to improve the efficacy of CAR-T cell therapy for viral diseases, cancer, and autoimmune diseases

**DOI:** 10.1186/s13287-026-05266-0

**Published:** 2026-09-06

**Authors:** Hany E. Marei, Giacomo Pozzoli, Sara Caratelli, Carlo Cenciarelli

**Affiliations:** 1 Department of Cytology and Histology, Faculty of Veterinary Medicine, Mansoura University, Mansoura, 35116 Egypt; 2 Institute of Pharmacology, Università Cattolica del Sacro Cuore, Rome, Italy; 3 Pharmacology Unit, Fondazione Policlinico Universitario A. Gemelli IRCCS, Rome, Italy; 4 Institute of Translational Pharmacology (IFT), National Research Council (CNR), Rome, Italy

**Keywords:** Chimeric Antigen Receptor (CAR) T cells, Next-generation CAR design and engineering, Gene editing and synthetic immunology, Tumor microenvironment resistance and cellular fitness, Universal, Allogeneic and, Immune tolerance–engineered CAR-T therapies

## Abstract

Chimeric antigen receptor (CAR)-T cell therapy has transformed the treatment of hematological malignancies, yet its broader application to solid tumors, chronic viral infections, and autoimmune diseases remains constrained by antigen heterogeneity, immunosuppressive tissue microenvironments, T-cell exhaustion, limited persistence, and treatment-associated toxicities. These challenges have shifted the field from optimizing individual receptor constructs toward engineering CAR-T cells as programmable immune systems capable of adapting to diverse disease contexts. This review synthesizes recent advances in molecular engineering strategies that enhance CAR-T cell function beyond conventional receptor design. We discuss how receptor engineering, genome editing, transcriptional and epigenetic regulation, metabolic reprogramming, synthetic gene circuits, and safety-control platforms collectively reshape CAR-T cell fate, persistence, and therapeutic efficacy. Rather than functioning independently, these engineering strategies are increasingly integrated to generate context-specific cellular therapies capable of adapting to diverse disease environments, including cancer, autoimmune diseases, and chronic viral infections. We also highlight the potential for translation into clinical practice or clinical translation and discuss the major challenges associated with clinical implementation. Next-generation CAR-T therapies will increasingly integrate molecular engineering strategies or will rely on molecular engineering strategies to integrate antigen recognition, cellular fitness, immune regulation, and longevity rather than simply maximizing cytotoxic activity. Recent advances in programmable cellular engineering coupled with rigorous clinical evaluation as well as scalable manufacturing technologies or scalable manufacturing platforms in the treatment of other diseases beyond oncology will facilitate the development of safer, more durable, and broadly applicable cellular therapies.

## Clinical trial number

Not applicable.

## Introduction — Why new molecular strategies matter now

The success of CD19 and BCMA-targeted CAR T cell therapy has proved that… or Clinical studies conducted on … have clearly shown that T cells are capable of treating some hematologic malignancies. Long-term remissions achieved in the previously incurable B cell malignancies provide evidence that gene-modified T cells can have durable anti-tumor activity, supporting the idea of vaccine cell therapy as a novel oncology treatment strategy [1]. Nonetheless, this first success also shows that a rather important biological law is observed: the enduring effect of CAR-T therapy does depend not only on the receptor design but also on the atmosphere in which modified T-cells act [1, 2].

Expanding CAR-T therapy applications in solid tumors is more complicated, with several obstacles to be dealt with, such as poor tumor trafficking, complexities of stromal tissue, the presence of cells that suppress T cell immunity, the presence of constant stimulation with target antigens leading to exhaustion, and the presence of competition for nutrition and other metabolites among cells within the tumor microenvironment [3, 4]. The presence of antigen reservoirs in chronic viral infections necessitates immune surveillance over prolonged periods without T cell exhaustion from engagement with antigenic elements, whereas patients with autoimmune disorders should be treated by eliminating infectious immune cells [5, 6].

This implies that the field has moved away from thinking of CARs simply as artificial antigen receptors and instead has created programmable immune cells, whose function can be manipulated by using receptor engineering, the genome, transcription, and epigenetics, metabolism, and synthetic genes. Consequently, instead of different engineering methods, the approaches being combined will enable the control of T-cell fate, survival, functioning, and safety in various settings [7, 8]. This change in approach—from purely improving receptors to engineering at the system level—forms the basis of the current review.

New research from laboratory experiments and earlier clinical trials indicates that long-lasting responses from CAR-T immunotherapy relate not only to engineering at the cell level but also to interactions between CAR-T cells and the immune system, including antigen-presenting cells, connective tissues, and endogenous lymphocyte populations. Findings support the idea that the reorganization of the immune system contributes to the prolonged effectiveness of CAR-T therapy; however, the precise mechanisms underlying this remain to be established in future experiments [9].

For this reason, the Review pays special attention to current evidence while distinguishing established processes from emerging theories. In cases where the evidence is preliminary, the authors assess such theories as potential areas for further research rather than as unequivocal biological facts.

**Fig. 1:**
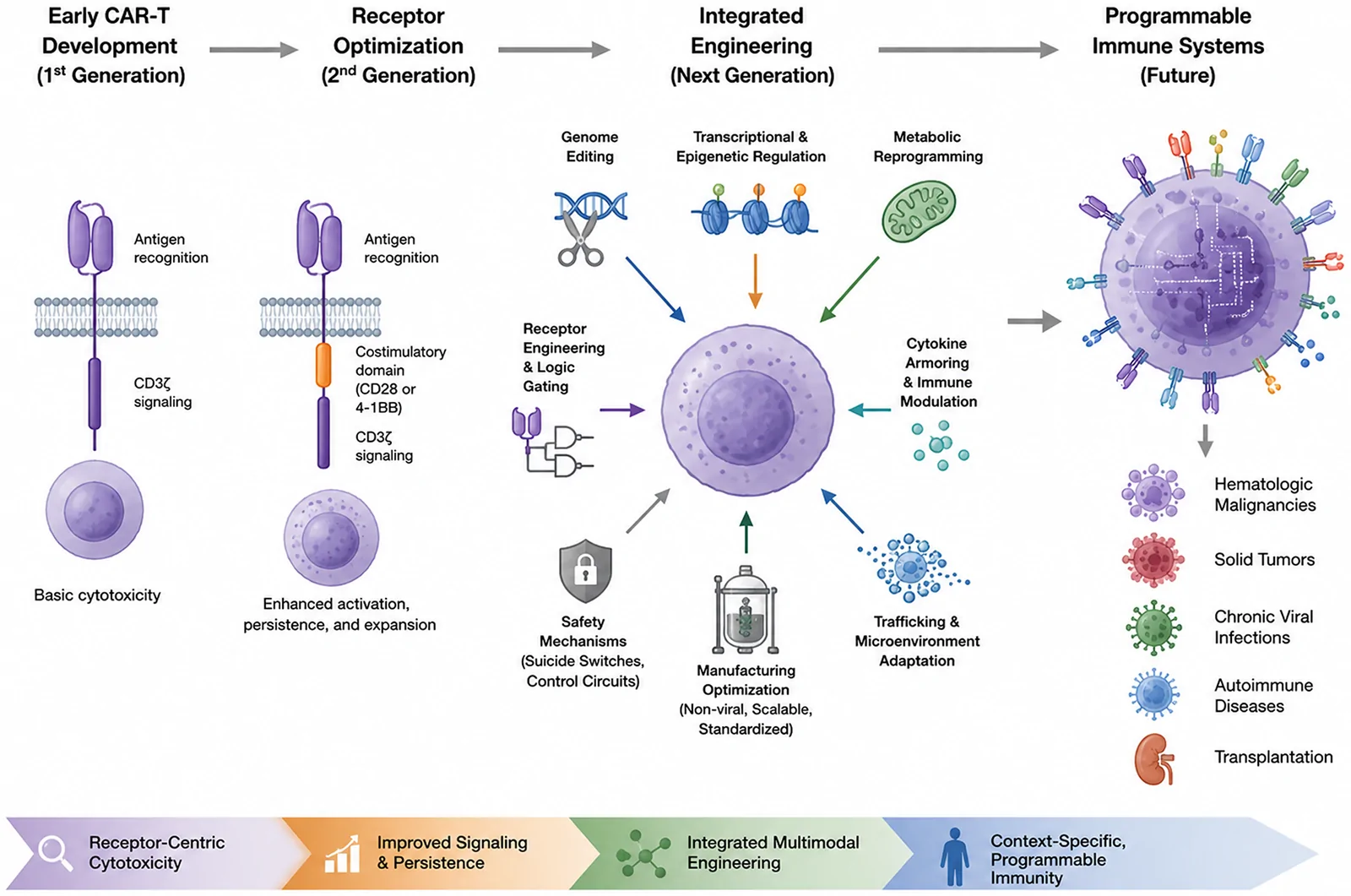
Evolution of CAR-T engineering from receptor optimization to programmable immune systems. Early CAR-T cell development focused primarily on optimizing CAR architecture and enhancing antigen recognition. In contrast, current engineering strategies extend beyond CAR design to incorporate genome engineering, transcriptional regulation, and metabolic reprogramming of immune cells. Together, these advances are enabling the development of engineered immune cells capable of dynamically modulating their function according to the biological context of different diseases. *This conceptual framework was developed based on previous studies describing the evolution of CAR-T cell engineering*,* synthetic immunology*,* genome engineering*,* and systems-level regulation of T-cell function by June and Sadelain (2018)*,* Gross et al. (1989)*,* Sadelain (2020)*,* Depil et al. (2020)*,* and Lim and June (2017)*

These developments indicate a fundamental shift in the engineering of CAR-T cells. Instead of using receptor signaling to achieve stronger cytotoxic effects than before, current approaches aim to optimize T cell engineering to combine antigen recognition with metabolic fitness, differentiation state, tissue adaptation, and immune regulation. This explains why we will organize the following sections according to a unified conceptual framework based on biological principles most important for clinical translation (Fig. 1).

## Molecular engineering of the car molecule and receptor logic

### Next-generation CAR architectures

The development of the CAR architecture reflects a broader shift from static antigen recognition to tunable immune detection. The first CARs improved T cell stimulation by adding costimulatory regions. In modern CAR designs, new functions are introduced that not only improve antigen specificity but also address issues such as minimizing off-tumor toxicity, reducing variability, and regulating CAR-T cell function. Rather than therapeutic failure programmable control of representing independent innovations, affinity optimization strategies, dual antigen recognition, advanced logic receptors, and adjustable CAR technologies collectively aim—to enable engineered T cells to respond to complex antigenic landscapes appropriately while promoting T-cell effector responses that are effective, safe, and durable.

Improving the hinge and transmembrane regions of the CAR, both of which are critical for structural integrity and receptor expression, further optimizes receptor stability and function. Finally, optimal tuning of the CAR co-stimulatory domains (e.g., CD28 or 4-1BB) is designed to balance rapid activation and long-term persistence while preserving effector function [10, 11].

Designing multispecific CAR technologies that simultaneously target multiple antigens is an approach to overcoming antigen escape as well as tumor heterogeneity. These technologies include tandem, bicistronic, dual, and logic-gated CARs, and they aim to improve tumor detection while limiting immune evasion due to antigen loss or heterogeneous antigen expression. Furthermore, besides expanding the spectrum of target antigens, multispecific receptor technologies may enhance T-cell activation by integrating signaling from receptors that recognize multiple antigens. However, while initial clinical trials and preclinical studies show promising results in terms of treatment effectiveness against hematological diseases, the increasing complexity of CAR platforms introduces additional challenges related to both manufacturing and potential biocompatibility, which require validation in clinical studies [12, 13].

More advanced “split” or universal CAR systems enable precise, externally controllable activation, thereby reducing potential off-tumor cytotoxicity. Combinatorial architectures, such as bispecific CARs and AND-gate CAR designs, enhance tumor selectivity by requiring simultaneous recognition of two antigens for CAR activation, while minimizing collateral damage to other tissues. A 2024 preclinical study of a modular ‘switch’ CAR T cell platform demonstrated that CAR T cells can be engineered to express a standard CAR in conjunction with a switch/yoke molecule derived from the natural ligands of APRIL or BAFF, enabling programmable modulation of CAR T cells using multiple adapter molecules [14]. This research showed that the use of a switch/yoked CAR enabled CAR T cells to recognize multiple tumor antigens and compensate for antigen loss, thereby broadening mechanisms of tumor recognition and elimination. The switch/yoke functioned as an effective safety mechanism limiting cytotoxic activity by CAR T cells on healthy tissues [14]. The switch technology elicited robust cytokine responses in preclinical studies, with the CAR T cells effectively eliminating tumors in murine models with various malignancies of B-cell origin despite reduced expression of target antigens [14, 15]. In addition, in 2024, Shi et al. confirmed that switch-based CAR T cells demonstrated clinical feasibility when used in conjunction with a bispecific CAR T cell therapy targeting BCMA and CD19 [16]. Such findings support the use of switch-based and/or bispecific CAR T cells to broaden the therapeutic potential of CAR T cell therapy by increasing CAR-T cell selectivity against malignant tumors, overcoming downregulation or heterogeneous expression of tumor antigens, and reducing the risk of collateral tissue damage.

Affinity engineering facilitates identification of normal and cancerous tissues by establishing signaling thresholds that rely on tumor antigen density, enhancing tumor specificity while minimizing target-associated toxicity [17, 18]. At the same time, adapter-based CAR architecture and logical gating provide additional levels of spatial and temporal regulation of T-cell activation, demonstrating that modern receptor engineering tends to focus on precise antigen discrimination and safety rather than simply enhancing receptor signaling [19, 20]. In addition to receptor design, the emerging field of T-cell molecular profiling has informed CAR engineering strategies.

Recent developments in the fields of single-cell transcriptomics, profiling of T-cell receptor (TCR) repertoires, and high-dimensional immune phenotyping have substantially advanced our understanding of CAR-T cells with respect to differentiation states, functional variability, and molecular mechanisms underlying therapeutic efficacy, persistence, side effects, and failure [21, 22].

Our understanding of the molecular determinants of CAR-T cell function has advanced substantially due to advancements in single-cell transcriptomics, profiling of the T-cell receptor (TCR) repertoire, and high-dimensional immune phenotyping. These technologies have enabled comprehensive characterization of T-cell differentiation states, clonal diversity, functional heterogeneity, and exhaustion programs, enabling identification of cellular features that underlie therapeutic efficacy, durability, and resistance [21]. Combined use of these profiling techniques with suitable models, such as patient-derived organoids and ex vivo immune systems, further enhanced these analyses [23].

### Switchable, universal, and transient CAR formats

Considerable efforts have focused on developing CAR architectures to enhance flexibility in controlling T-cell activation, antigen specificity, and persistence. Multiple molecular approaches now enable reversible, dose-dependent control of CAR signaling. Drug-inducible dimerization domains and protease-cleavable safety switches are representative small-molecule-gated systems that enable transient activation or deactivation of CAR-T cells in vivo, reducing the risk of cytokine release syndrome (CRS) or off-target toxicity [24–27].

Universal adapter CARs are another major advance in CAR technology, using a universal receptor scaffold (often an scFv that binds an inert peptide epitope, such as GCN4) to recognize tumor cells only in the presence of the bispecific adaptor protein. The modularity of this design enables rapid retargeting of CARs to other antigens without requiring complete redesign of the CAR-T immunotherapy product, while also providing more precise dose-dependent regulation by modulating the amount of the adaptor protein present [19, 28–34].

Despite the encouraging antitumor activity of adapter-controlled CAR technologies observed in preclinical studies in hematological and solid tumors, a major advantage of this platform is that it enables reversible, dose-dependent control of CAR-T cell activation, which may enhance efficacy and safety [35].

The unique approach of the Universal CAR platform technology involves transient CAR expression enabled by mRNA delivery techniques. This method allows for transient therapeutic applications in cases where sustained CAR expression may be undesirable. Clinical research involving various CAR-T therapies targeting mesothelin and HIV envelope antigens demonstrated promising antitumor activity and achieved good antiviral outcomes. In general, new technologies for CAR-T cells are transforming in the development of adoptive T-cell therapy and its use across various therapeutic settings [36–38].

Cotinine-based adapter systems exemplify inert molecular tags within universal CAR platforms that enable reversible, externally controlled CAR-T activation. This approach improves therapeutic precision and lowers the risk of off-tumor toxicity because cytotoxic activity is restricted when tumor-specific adapter molecules are present [19].

## Gene editing & precise genome engineering: enabling durable, safer CAR-T cells

### CRISPR-based disruption of endogenous immune programs for universal CAR-T manufacture

Genome editing has transformed CAR-T cell engineering, from introducing synthetic receptors to modifying intracellular pathways that govern immune compatibility, longevity, and safety of therapy. Beyond being a gene-editing tool, CRISPR-based engineering serves as a versatile platform for creating universal allogeneic CAR-T products by simultaneously disrupting signaling receptors in T-cells and mechanisms of host immune recognition. Therefore, genome editing is a central strategy for improving both biological performance and manufacturing feasibility of next-generation CAR-T therapies.

The CRISPR/Cas system is among the most significant advances in CAR T cell development. It enables targeted editing of the TRAC or TRBC locus, thereby eliminating the native TCR from CAR-T cells, preventing graft-versus-host disease and creating universal allogeneic CAR T products [8] In addition to editing TCR loci, deleting β2-microglobulin (β2M) removes HLA class I expression on the CAR T-cell surface, reducing host immune rejection and enhancing CAR T persistence in an allogeneic environment [39, 40]. CRISPR-based strategies enable efficient, simultaneous editing of multiple targets using simple-to-design guide RNAs, thereby facilitating rapid and reproducible manufacturing for CAR T cell products [41, 42]. The transient delivery of Cas9 or Cas12a ribonucleoprotein complexes minimizes prolonged nuclease exposure. It thus decreases the risk of off-target mutations, supporting clinical translation [43].

### Targeted knock-in strategies for controlled CAR expression and signaling fidelity

The targeted knock-in techniques represent a major advance over classical viral gene delivery methods; they enable precise insertion of CAR transgenes at predefined genomic loci. Insertion into the TRAC locus enables CAR expression under the control of native T cell receptor genes, ensuring uniform CAR expression, lower tonic signaling, and improved functional persistence, as well as reducing cell exhaustion compared with random viral insertion. These properties increase the safety and reproducibility of CAR-T products, providing a standardized platform for subsequent genome engineering.

Targeted integration of CAR constructs into the TRAC locus can be accomplished via either the CRISPR-Cas9 or Cas12a systems. Cas12a, in particular, provides several advantages over Cas9, including staggered DNA cleavage and higher efficiency for multiplex gene knock-in at the target site [44, 45]. Simultaneously disrupting the endogenous TCR signaling pathway and introducing a CAR cassette improves safety and therapeutic efficacy [8, 46]. Targeted integration of CAR constructs into the TRAC locus offers several advantages over traditional viral-mediated gene transfer, including reduced risk of insertional oncogenesis associated with semi-random viral integration, as demonstrated by studies reporting post-therapy clonal expansion in conventional CAR-T products [47, 48].

### Multiplex genome editing and molecular control of CAR-T cell function

CRISPR technology provides a versatile platform for developing a range of genome-editing methods that enable the simultaneous introduction of targeted knock-out and knock-in mutations in a single CAR-T product, thereby enhancing persistence, potency, safety, and programmable cellular functions. At the same time, the new pooled knock-in technologies enable high-throughput insertion of engineered genetic programs and systematic characterization of cellular phenotypes [49].

Simultaneous disruption of inhibitory immune checkpoints (such as PD-1, CTLA-4, or LAG-3) and CAR-T activation and cytotoxicity are enhanced by preventing adaptive immune suppression within the tumor microenvironment [39, 50, 51]. Additionally, incorporating CAR constructs into inhibitory loci (such as PDCD1) couples checkpoint disruption with stable CAR expression, thereby generating intrinsically armored CAR-T cells that are more durable and less exhausted [52]. The implementation of base editing and prime editing technologies further enhances genome-editing precision, as these technologies enable targeted nucleotide substitutions without inducing double-strand breaks, reducing chromosomal rearrangements and increasing the predictability of edits [53–56]. These technologies will enable controlled gene silencing via premature stop codons or splice-site disruption while maintaining genomic integrity, thereby providing a distinct advantage over multiplex editing technologies [55].

In summary, these advances show that genome-editing technology is evolving from a manufacturing tool to a platform for programmable control of CAR-T cell biology. Integrating targeted knock-in strategies, multiplex genome editing, and modern precision genome editing technologies enables the optimization of engineered lymphocytes for persistence, functional competence, resistance to exhaustion, and therapeutic safety. Further clinical studies will clarify whether integrated innovations can establish effective and broadly accessible cellular therapies.

Clinical evidence indicates that interactions between CAR-T cells and the host immune system are a key determinant of the success of CAR-T treatment. However, it remains unclear how well the CAR-T cells remodel the host immune system [57–59]. While persistence, expansion, and differentiation of CAR-T cells are associated with improved clinical outcomes, evidence supporting durable immune reconstitution following CAR-T therapy remains limited. Collectively, these findings suggest that CAR-T cells must be developed not only to enhance their cytotoxic capacity but also to promote coordinated interactions with the host immune system [60, 61].

Despite their considerable therapeutic potential, multiplex genome editing poses significant safety issues that require careful consideration before widespread clinical implementation. By making changes to many genomic sites, the risk of unintended genomic alterations increases, increasing the likelihood of adverse genomic events, such as chromosomal abnormalities, translocations, large genomic deletions, and clonal expansion, whereas the long-term consequences on cellular function, genomic stability, and malignant transformation remain unknown. These challenges underscore the need for developing more precise genome-editing technologies, comprehensive genomic surveillance, as well as long-term clinical follow-up of genome-edited CAR-T therapies [62–67].

Overall, current innovations in genome editing highlight a transition from single-gene modification to programmable regulation of CAR-T cell function. Future therapeutic strategies will likely rely on a combination of receptor engineering, site-specific gene modifications, and systems-level engineering approaches rather than isolated single-gene modifications.

## Overcoming inhibitory cytokines and the suppressive microenvironment (“Armoring”)

The efficacy of CAR-T cells in treating solid tumors is shaped by the immunosuppressive landscape of the tumor microenvironment (TME). The TME comprises immunosuppressive cytokines, immune checkpoint ligands, stromal barriers, and immunosuppressive myeloid populations, all of which collectively impair antitumor immune responses. Accordingly, armoring strategies are designed to enhance CAR-T cell therapeutic efficacy and remodel the local immune microenvironment by modulating cytokine signaling, immune checkpoint signaling, and cellular interactions within the tumor microenvironment [68, 69].

### Dominant-negative receptors and cytokine decoys

One of the most effective strategies to overcome TME-mediated immunosuppression is to disrupt CAR-T cell responses to inhibitory cytokines, such as transforming growth factor-β (TGF-β). Among the immunosuppressive mediators within the TME, TGF-β represents one of the principal barriers to CAR-T cell proliferation, cytotoxic function, and persistence in solid tumors. These findings have driven the development of dominant-negative TGF-β receptors (dnTGF-βRs) as an effective strategy to uncouple CAR-T cell function from suppressive cytokine signaling while preserving CAR-T cell activity within immunosuppressive tumor microenvironments.

Expression of dominant-negative TGF-β receptors (dnTGF-βRs) in CAR-T cells renders CAR-T cell function resistant to ambient TGF-β signaling, thereby preserving sustained proliferation and effector function within immunosuppressive tumor microenvironments. Consequently, preclinical studies across multiple solid tumor models have demonstrated enhanced CAR-T cell proliferation, effector cytokine production, and antitumor activity with dnTGF-βR-expressing CAR-T cells compared with unmodified CAR-T cells [70, 71]. Early clinical studies support the feasibility, safety, and continued clinical development of this approach. In addition to TGF-β inhibition, dominant-negative and decoy receptor strategies represent a complementary approach to armored CAR-T cell engineering, in which selective blockade of suppressive signaling pathways may enhance resistance to immune suppression without relying solely on enhanced T-cell activation. Furthermore, whether analogous strategies targeting additional inhibitory cytokines can achieve comparable efficacy and safety remains an important direction for future research.

### Cytokine armoring and localized immune modulation

While dominant-negative receptors confer resistance to immunosuppressive signaling, cytokine armoring actively remodels the tumor microenvironment by promoting localized immune activation. Engineering CAR-T cells to secrete cytokines such as IL-12 and IL-18 enhances recruitment and activation of innate and adaptive immune cells while minimizing the systemic toxicities associated with recombinant cytokine therapy through localized cytokine release within the tumor microenvironment [68].

Furthermore, IL-12-armored CAR-T cells demonstrate that enhanced IL-12 secretion drives extensive remodeling of the tumor microenvironment, bystander activation of immune cells, and epitope spreading beyond the initially targeted antigens. A recent study evaluated localized IL-12 delivery in combination with PD-L1 targeting, demonstrating that multifunctional armored CAR-T cell platforms promote antitumor immunity while reducing systemic toxicity [68].

Cytokine armoring is associated with several important limitations. Sustained IL-12 signaling drives terminal effector differentiation of T cells, activation-induced cell death (AICD), and the accumulation and activation of myeloid-derived suppressor cells (MDSCs), thereby limiting long-term CAR-T cell persistence and increasing the risk of tissue injury [72, 73]. Similar to other IL-15-based armoring approaches, IL-15-mediated maintenance of memory T-cell states imposes substantial metabolic demands that may be poorly suited to the nutrient-deprived tumor microenvironment. Therefore, cytokine armoring may reprogram T-cell differentiation to optimize CAR-T cell function rather than merely augmenting effector function [74].

Beyond its direct effects on CAR-T cells, cytokine armoring also reshapes the surrounding immune microenvironment. IL-7/CCL19-armored CAR-T cells not only recruit dendritic cells and naïve T cells but also promote the formation of tertiary lymphoid structures within tumors and establish organized immune niches within the local tissue microenvironment [75]. Collectively, these findings suggest that armored CAR-T cells function as local orchestrators of antitumor immunity rather than simply as autonomous cytotoxic effectors.

### Checkpoint modulation built into CAR-T cells

Immune checkpoint pathways are major drivers of CAR-T cell dysfunction within the tumor microenvironment, with the PD-1/PD-L1 axis representing one of the most extensively studied mechanisms of resistance. Recent advances in CAR-T cell engineering have enabled localized modulation of immune checkpoint signaling through the incorporation of PD-1/PD-L1-blocking antibodies, dominant-negative PD-1 variants, or switch receptors that convert inhibitory signals into activating signals [3, 76].

By enabling localized secretion of scFvs or expression of intracellular dominant-negative checkpoint receptors, these platforms further enhance CAR-T cell function by blocking inhibitory signaling within the tumor microenvironment, thereby sustaining T-cell effector function and enhancing antitumor activity in preclinical models [68, 77] Recent advances in immunology indicate that immune checkpoint pathways should not be viewed solely as inhibitory regulators. For instance, PD-1 contributes to the maintenance of T-cell survival, memory T-cell differentiation, and adaptation to repeated antigen exposure. Therefore, complete PD-1 blockade may accelerate T-cell loss while impairing long-term T-cell persistence [78].

Collectively, these observations suggest that future checkpoint-armored CAR-T cells may increasingly incorporate conditional or tunable checkpoint modulation rather than constitutive checkpoint blockade. Such strategies may preserve the physiological functions of checkpoint signaling in memory T-cell differentiation and immune homeostasis while minimizing tumor-induced immunosuppression, thereby improving both the durability and safety of CAR-T cell therapy [78].

Various armoring strategies redefine CAR-T cells as active architects of the immune microenvironment beyond their conventional cytotoxic functions. By reprogramming their responses to cytokine signaling, reshaping local inflammatory gradients, and modulating immune checkpoint signaling, armored CAR-T cells engage fundamental mechanisms of immunoregulation, spatial immune organization, and tissue adaptation. Moving forward, the most critical challenge will be to integrate these engineering strategies with an improved understanding of immune homeostasis to ensure that enhanced antitumor efficacy is not achieved at the expense of durability, adaptability, and safety [79, 80].

## Preventing and reversing exhaustion — transcriptional and epigenetic reprogramming

Rather than being viewed as an irreversible state of cellular dysfunction, T-cell exhaustion is now recognized as a distinct differentiation program characterized by unique chromatin and transcriptional states that can be therapeutically reprogrammed through targeted engineering [6, 81, 82].

### Transcription factor manipulation (c-Jun, BATF/IRF4, TOX/NR4A pathways)

The recognition that sustained dysregulation of transcription factor networks downstream of TCR signaling contributes to T-cell exhaustion has represented a major conceptual advance in the field. Chronic antigen stimulation promotes sustained NFAT activation in the absence of adequate AP-1 cooperation, thereby inducing the expression of exhaustion-associated transcription factors, including TOX and members of the NR4A family, which collectively establish a transcriptional program that limits durable effector function [83, 84].

A single transcriptional modification reduced inhibitory receptor expression, preserved cytokine production, and enhanced antitumor efficacy across multiple preclinical models. Collectively, the ability to reprogram T-cell fate through targeted modulation of intrinsic signaling pathways provides a strong rationale for the early clinical evaluation of this approach.

Recent studies have demonstrated that BATF and IRF4 function as context-dependent regulators rather than universal drivers of T-cell exhaustion. Their effects depend on the strength and duration of antigen stimulation, enabling them to promote either sustained effector function or terminal differentiation depending on the signaling context [85].

This study highlighted the importance of balancing dynamic transcriptional programs governing effector T-cell differentiation while providing evidence that, although many transcriptional regulators are shared across multiple T-cell states, TOX and members of the NR4A family emerge as the principal regulators of the exhausted T-cell phenotype.

Genetic and epigenomic studies have shown that TOX and TOX2 cooperate with members of the NR4A family to establish and maintain the exhausted T-cell state through chromatin remodeling, thereby driving the expression of inhibitory receptors characteristic of T-cell exhaustion [84, 86]. Genetic suppression or deletion of these factors has been shown to partially restore T-cell proliferation and effector function in CAR-T cells, confirming that the exhaustion program is amenable to therapeutic modulation. Furthermore, recent studies have demonstrated that T-cell exhaustion comprises multiple distinct differentiation states organized along a developmental continuum. A reservoir of TCF1-expressing progenitor exhausted (T < sub> PEX</sub>) cells retains epigenetic plasticity and serves as a self-renewing population capable of proliferating and generating terminally exhausted progeny following therapeutic reinvigoration [87, 88]. These findings suggest that transcriptional engineering strategies are likely to be most effective when implemented before exhaustion-associated chromatin remodeling becomes epigenetically fixed.

### Epigenetic and chromatin-level editing

Manipulating transcription factors can confer substantial functional benefits; however, these benefits may be progressively attenuated as exhaustion-associated epigenetic remodeling becomes established. Exhausted T cells acquire stable enhancer landscapes enriched at genomic regions bound by TOX and members of the NR4A family, accompanied by reduced chromatin accessibility at memory-associated loci regulated by TCF1 and LEF1 [89, 90]. Exhaustion-associated chromatin landscapes persist despite antigen withdrawal and following immune checkpoint blockade, highlighting the intrinsic epigenetic barrier to T-cell reinvigoration. More recent studies have demonstrated that the principal barrier to durable functional recovery is the epigenetic fixation of the exhausted state rather than transient transcriptional dysregulation.

Chronic antigen stimulation progressively drives stable chromatin remodeling, establishing gene expression programs characteristic of T-cell exhaustion and thereby limiting cellular plasticity. Consequently, successful functional restoration requires remodeling of these epigenetic programs rather than relying solely on transient transcriptional reprogramming [91].

Current engineering strategies do not aim to eliminate dysfunctional cellular programs entirely but instead seek to preserve cellular function and restore epigenetic plasticity. One such strategy involves the genetic manipulation of chromatin-regulating factors or transient exposure to agents that promote stem cell–like properties [92, 93].

## Metabolic reprogramming — fueling sustained function in hostile niches

CAR-T cells function within metabolically hostile microenvironments characterized by hypoxia, nutrient deprivation, and the accumulation of metabolic byproducts, all of which impair mitochondrial function and promote T-cell exhaustion. Consequently, metabolic fitness is a critical determinant of CAR-T cell persistence, differentiation, and cytotoxic function. Beyond supplying cellular energy, metabolism regulates gene expression and cellular programs governing T-cell fate decisions, making metabolic engineering an increasingly attractive therapeutic strategy [94, 95].

Studies have demonstrated that T-cell metabolism directly regulates T-cell differentiation and exhaustion. Nutrient availability, mitochondrial activity, and mitochondria-derived redox metabolites are closely linked to transcriptional networks governing T-cell activation. Similar to transcriptional and epigenetic regulation, immunometabolism represents a key determinant of T-cell fate decisions [96, 97].

### Microenvironmental metabolic cues and T-cell fitness

CAR-T cell function is profoundly influenced by the tumor microenvironment, where altered ionic and metabolic cues restrict nutrient availability, impair mitochondrial function, and shape T-cell differentiation. T-cell function is further compromised by nutrient deprivation under metabolic stress induced by factors such as hypoxia, ionic dysregulation, and competition for essential nutrients [98].

Lactate metabolism exemplifies this metabolic complexity. Although metabolic adaptation can promote T-cell survival in nutrient-deprived microenvironments, excessive reliance on MCT1-mediated lactate uptake can promote T-cell exhaustion, highlighting that metabolic substrate transport is critical for sustaining CAR-T cell function [99, 100].

### Mitochondrial fitness, redox balance, and longevity

Mitochondrial integrity is a defining feature of durable T-cell immunity. Both memory T cells and stem cell-like T cells rely predominantly on oxidative phosphorylation and exhibit greater spare respiratory capacity than short-lived effectors or exhausted T cells. Enhancing mitochondrial biogenesis through regulators such as PGC-1α promotes metabolic adaptability under chronic antigen stimulation by preserving mitochondrial respiratory capacity, resistance to oxidative stress, and cell survival despite hypoxia and nutrient deprivation [101, 102].

Importantly, PGC-1α upregulation does not merely enhance effector function but also promotes a metabolic program that supports long-term persistence, thereby aligning the metabolic profile of CAR-T cells with that of endogenous memory T cells [96].

### Engineering nutrient acquisition and alternative fuel utilization

Engineering nutrient acquisition has emerged as an important strategy for maintaining CAR-T cell function within nutrient-deprived tumor microenvironments. Enhanced GLUT1 expression improves metabolic flexibility, proliferation, cytokine production, and antitumor activity. In addition, studies of lactate metabolism suggest that selective substrate utilization is more effective than indiscriminate enhancement of alternative metabolic pathways [103, 104].

### Manufacturing interventions and metabolic imprinting

Metabolic programming is initiated during ex vivo manufacturing, as signaling cues and the cytokine milieu shape long-term cellular function. Ex vivo expansion in the presence of IL-7 and IL-15, together with transient modulation of mTOR signaling, promotes stem-like and central memory T-cell phenotypes with enhanced mitochondrial fitness, demonstrating that manufacturing conditions establish durable metabolic programs before infusion [96, 101].

### Metabolic trade-offs and epigenetic feedback

Because excessive reliance on glycolytic or mitochondrial metabolism may promote terminal differentiation, oxidative stress, and loss of self-renewal capacity, metabolic reprogramming must be precisely regulated. Therefore, successful metabolic engineering strategies should aim to optimize rather than maximize metabolic activity to sustain long-term CAR-T cell persistence and function [101, 104].

These trade-offs underscore the importance of precise metabolic engineering. In addition, metabolism and epigenetic regulation are tightly interconnected. Metabolites such as acetyl-CoA, α-ketoglutarate, and S-adenosylmethionine serve as essential substrates and cofactors for chromatin-modifying enzymes, thereby directly linking nutrient availability to chromatin accessibility. Consequently, chronic metabolic stress can reinforce the epigenetic landscape associated with T-cell exhaustion. Conversely, early metabolic support can preserve an epigenetically permissive state and maintain developmental plasticity [96]. As such, metabolic interventions should be used in conjunction with epigenetic interventions for long-term functional rescue.

**Fig. 2:**
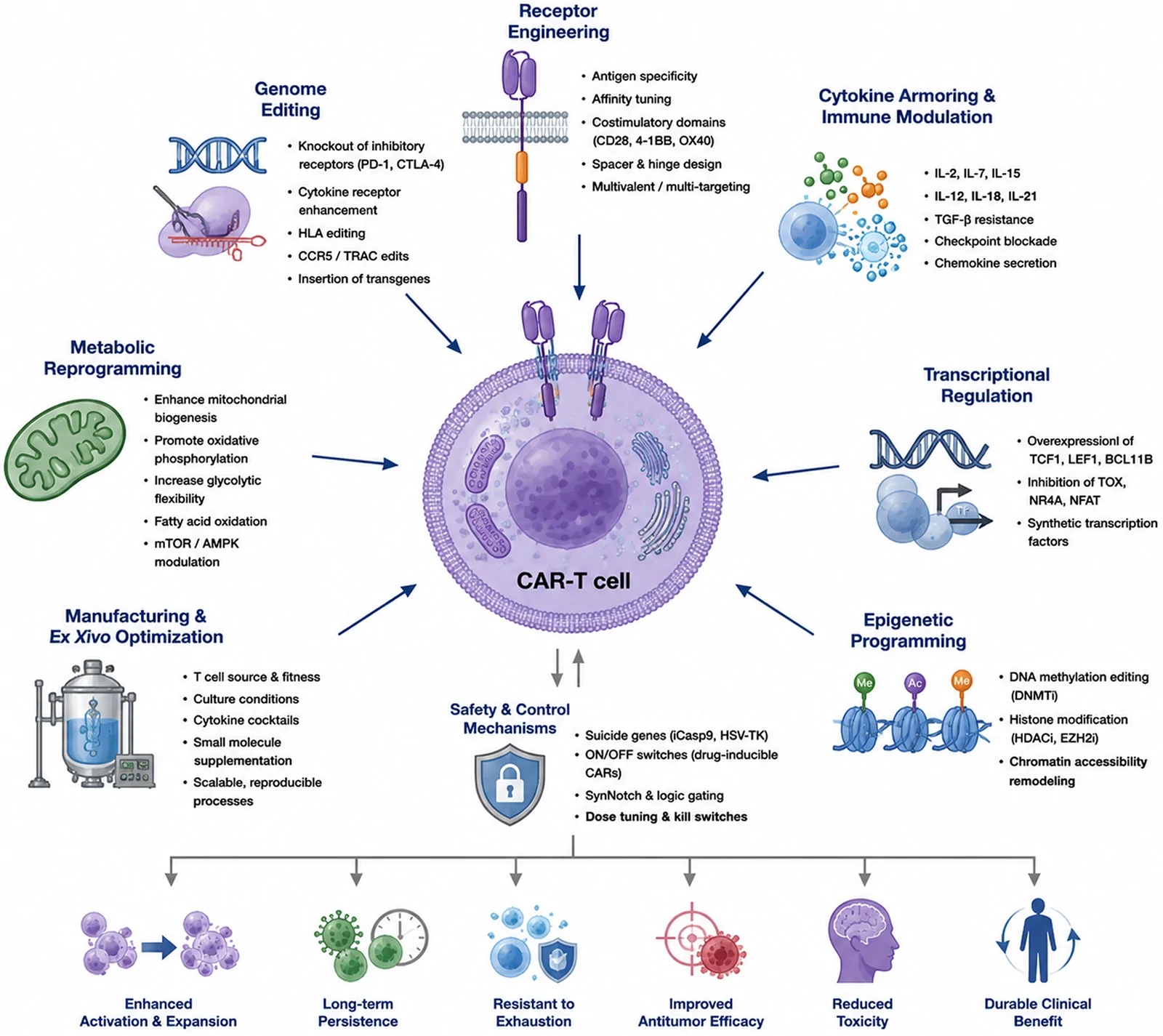
Integrated molecular determinants of CAR-T-cell function. Multiple molecular engineering strategies have been developed to optimize CAR-T cell function and therapeutic efficacy. These include CAR engineering, genome engineering, cytokine armoring, transcriptional and epigenetic programming, metabolic reprogramming, and optimized CAR-T cell manufacturing. Collectively, these strategies regulate the function and therapeutic performance of CAR-T cells by influencing activation, persistence, differentiation, resistance to exhaustion, metabolic fitness, and safety. *This conceptual framework was developed based on previous studies investigating CAR engineering*,* genome engineering*,* T-cell exhaustion*,* immunometabolism*,* cytokine armoring*,* and CAR-T cell persistence*,* as reported by Lim and June (2017)*,* Fraietta et al. (2018)*,* Blank et al. (2019)*,* Buck et al. (2017)*,* and Ho and Kaech (2022)*

In summary, metabolic engineering has evolved from enhancing cellular bioenergetics to directing CAR-T cell differentiation and fate. Future strategies are expected to integrate metabolic reprogramming with transcriptional engineering, epigenetic modulation, and receptor engineering to generate CAR-T cells with enhanced metabolic fitness, durable persistence, and sustained antitumor function (Fig. 2) (Table 1).

**Table 1:** Molecular logic governing CAR-T cell function across disease contexts

Dimension	Cancer	Viral diseases	Autoimmune diseases	Key references
Therapeutic goal	Sustained elimination of malignant cells with controlled persistence	Durable suppression or reduction of viral reservoirs	Restoration of immune tolerance or immune repertoire “reset.”	[4, 105, 106]
Antigen biology	High but heterogeneous antigen density; frequent antigen loss	Low-density, intermittent or latency-associated viral antigens	Self or lineage antigens; often broadly or dynamically expressed	[107–109]
CAR activation threshold	Tuned to discriminate tumor from normal tissue	Lower sensitivity is required to detect a sparse antigen	Constrained signaling to avoid bystander or systemic activation	[110–112]
Receptor logic & control	Dual/multiplex CARs, AND gates, switchable/adaptor systems	High-sensitivity CARs, multiplex targeting, transient expression	Signal-dampened CARs (CAR-Tregs) or lineage-depleting CARs	[113–115]
Dominant suppressive pressures	TGF-β, PD-1 ligands, suppressive myeloid networks	Chronic stimulation, immune evasion, activation-induced cell death	Pro-inflammatory cytokines destabilize tolerance	[5, 116, 117]
Armoring strategies	dnTGF-βR, cytokine delivery (IL-12/IL-15), checkpoint modulation	Resistance to immune clearance; metabolic and survival support	Epigenetic and transcriptional reinforcement of regulatory identity	[118–120]
Transcriptional fate control	Balancing effector function and exhaustion (TOX, NR4A, c-Jun)	Preservation of progenitor-exhausted states	FOXP3-centered regulatory programs	[85, 121, 122]
Epigenetic constraints	Fixation of exhaustion-associated enhancer landscapes	Early epigenetic commitment limits reinvigoration	Maintenance of Treg-specific chromatin (FOXP3 TSDR)	[78, 123]
Metabolic requirements	Mitochondrial fitness and oxidative metabolism in nutrient-poor TME	Metabolic resilience under chronic stress	Metabolic restraint supporting suppressive stability	[74, 124, 125]
Desired persistence profile	Long-lived, memory-like CAR-T cells	Sustained but regulated surveillance	Long-lived CAR-Tregs or transient cytotoxic CAR-T	[126, 127]
Principal safety risks	CRS, neurotoxicity, off-tumor cytotoxicity	Immune escape, selection pressure	Global immunosuppression or loss of tolerance specificity	[128, 129]
Conceptual CAR-T model	Programmable cytotoxic immune modulator	Synthetic immune surveillance system	Engineered tolerance enforcer or immune reset platform	[20, 130]

## CAR-T strategies tailored to viral diseases

**Fig. 3:**
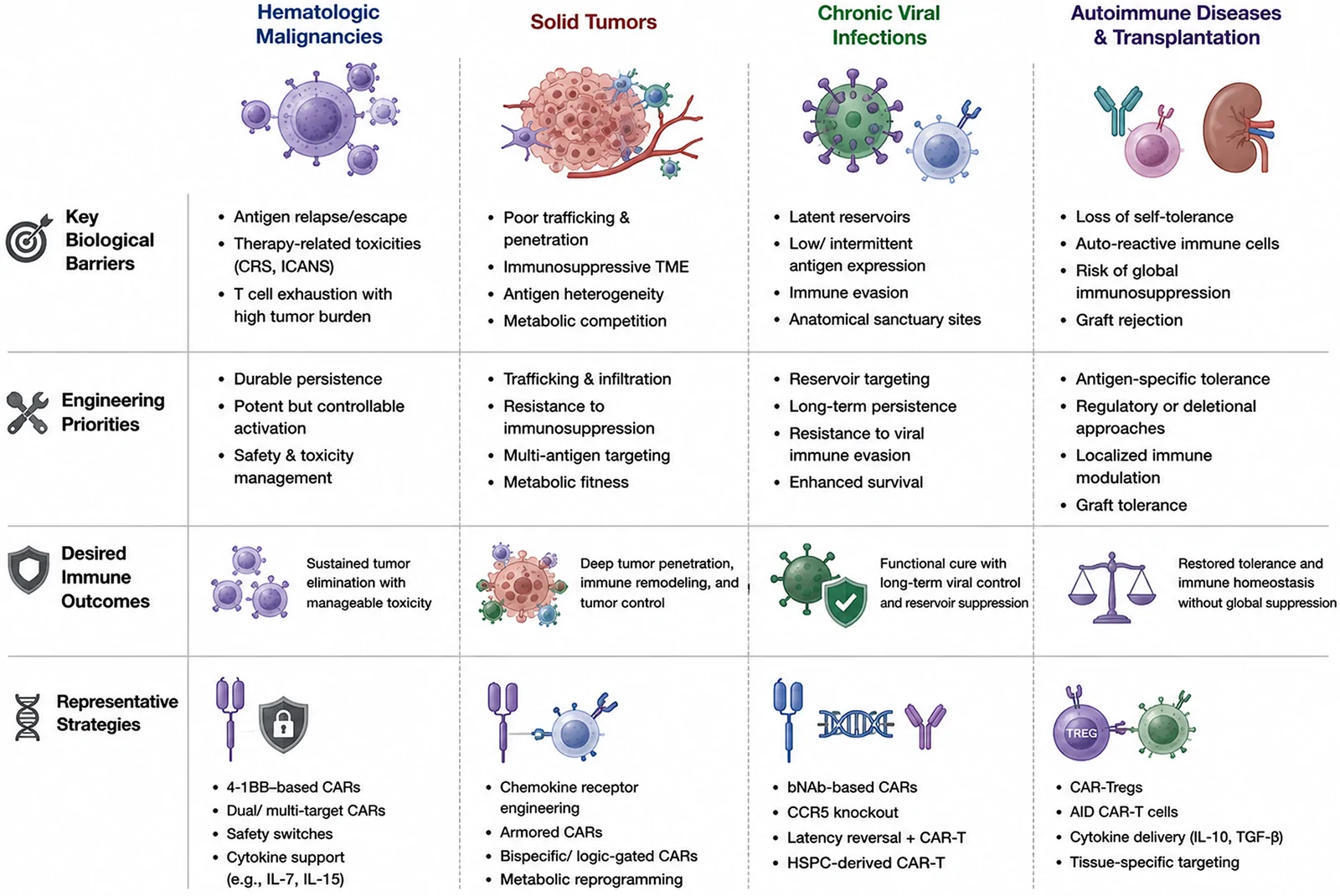
Disease-specific barriers and corresponding CAR-T engineering strategies. Distinct disease settings present unique biological challenges that require disease-specific CAR-T cell engineering strategies. In hematological malignancies, engineering efforts should prioritize improving safety and enhancing CAR-T cell persistence, whereas enhancing CAR-T cell trafficking and overcoming the immunosuppressive tumor microenvironment are critical for the treatment of solid tumors. In chronic viral infections, durable immune surveillance, effective targeting of viral reservoirs, and prevention of viral immune escape are essential. In contrast, therapeutic strategies for autoimmune diseases and transplantation should focus on promoting immune regulation and re-establishing antigen-specific tolerance. *This conceptual framework was developed based on previous studies by [Author(s)] describing disease-specific barriers to CAR-T cell therapy*,* tumor immunology*,* mechanisms of chronic viral persistence*,* and immune tolerance Chen and Mellman (2017)*,* Newick et al. (2017)*,* Wherry and Kurachi (2015)*,* Bluestone and Tang (2018)*,* and June and Sadelain (2018)*

The application of CAR-T cell therapy to chronic viral infections represents a significant expansion of cellular immunotherapy beyond its established role in oncology. Unlike tumors, chronic viral infections establish long-lived cellular reservoirs and drive persistent or intermittent antigen exposure while promoting immune evasion and anatomical sequestration, collectively impairing immune-mediated clearance. Consequently, virus-specific CAR-T cell therapy must not only eliminate infected cells but also recognize virally expressed antigens, traffic to tissue-resident viral reservoirs, withstand chronic immunosuppressive signaling, and maintain long-term functional persistence. Despite encouraging results in preclinical studies and early-phase clinical trials, no current therapy can completely eradicate persistent viral reservoirs. Consequently, emerging engineering strategies increasingly integrate advances in virology, reservoir biology, and immunology to overcome these barriers [110, 131–137] (Fig. 3).

### Antigen selection and reservoir targeting

The use of CARs produced from bNAb has already been shown to work for reservoir targeting, but these CAEs are limited by antigen variability and latent viruses [110].

However, incomplete eradication of the HIV reservoir and the virus’s ability to rebound in patients treated with bNAb-matched CAR-T cells indicate limitations in using CAR-T cells against HIV-associated antigen heterogeneity and latency [118, 138].

Beyond viral envelop proteins, several emerging strategies are under investigation that target host-derived surface markers selectively expressed during viral infection or immune activation. A comprehensive understanding of HIV reservoir biology indicates that no single antigen can reliably identify every infected cell because of the cellular and anatomical heterogeneity of viral reservoirs [139]. Consequently, antigen selection is increasingly recognized as a dynamic process that may require multiplex CAR-T cell designs to achieve sufficient antigen coverage in combination with latency-reversal strategies that induce transient antigen expression at levels sufficient for CAR-T cell recognition and engagement. Similar challenges exist in HBV infection, where viral antigens such as HBsAg and HBcAg are expressed at highly variable levels across infected hepatocytes, raising concerns regarding target specificity and on-target, off-tumor toxicity. Collectively, these findings suggest that optimal antigen selection should consider not only target specificity but also the spatial, temporal, and phenotypic heterogeneity of viral reservoirs. Accordingly, combinatorial strategies are increasingly favored to enhance viral reservoir eradication [140–145].

### Engineering for latency and reservoir access

Even with optimal antigen selection, CAR-T cell therapies must overcome intrinsic anatomical, transcriptional, and immunological barriers that protect persistent viral reservoirs and limit therapeutic efficacy. During latency, infected cells exhibit minimal viral gene expression, resulting in limited presentation of viral peptide–MHC complexes for immune recognition and thereby imposing intrinsic constraints on immune-mediated clearance. Consequently, effective CAR-T cell therapy requires strategies that either reverse viral latency or circumvent its immunological consequences to enable efficient recognition and elimination of infected cells [139].

Current engineering strategies aim to enhance target cell recognition and elimination, improve sensitivity to low-density antigen expression, and facilitate the identification of infected cells within anatomically protected viral reservoirs through optimization of chemokine receptor expression and CAR design [146, 147].

Enhancing resistance to immunological stress represents another major focus of CAR-T cell engineering. Perica et al. demonstrated that incorporation of the HIV immune evasion protein Nef into allogeneic CAR-T cells enhanced in vivo persistence and sustained functional activity [148]. By reducing activation-induced cell death (AICD) and immune-mediated T-cell clearance, Nef incorporation enhanced CAR-T cell persistence under conditions of chronic immune activation characteristic of persistent viral infections. Accordingly, this strategy may represent a synthetic counterpart to the natural immune evasion mechanisms exploited by viruses to persist despite sustained host immune pressure [149, 150].

**Fig. 4:**
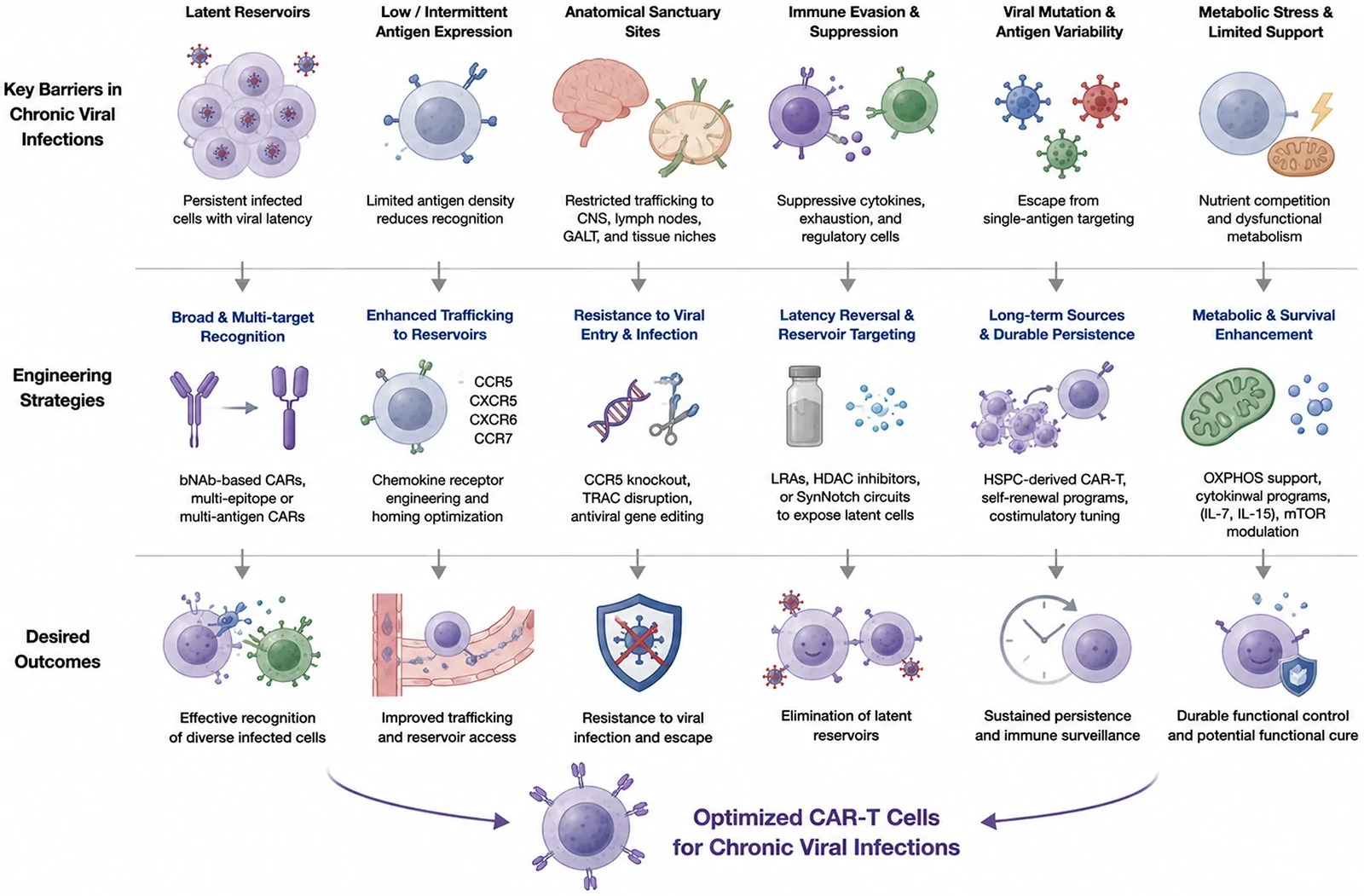
Engineering strategies for CAR-T-cell therapy against chronic viral infections. Chronic viral infections present multiple barriers to durable immune control, including persistent viral reservoirs, low or absent viral antigen expression, restricted immune access to anatomical reservoir sites, viral escape mutations, and chronic immune dysfunction. Current engineering strategies focus on broadly reactive antigen-targeting strategies, including broadly neutralizing antibody (bNAb)-based CARs, together with chemokine receptor engineering, CCR5-targeted genome engineering, hematopoietic stem cell-based CAR therapies targeting latent viral reservoirs, and approaches that enhance metabolic fitness. Collectively, these approaches aim to enhance CAR-T cell persistence, improve trafficking to viral reservoir sites, and sustain long-term immune surveillance. *This conceptual framework was developed based on previous studies by [Author(s)] describing CAR-T cell therapy for chronic viral infections*,* HIV reservoir biology*,* broadly neutralizing antibody (bNAb)-based CARs*,* and antiviral immune surveillance Deeks et al. (2021)*,* Zhen et al. (2017)*,* Leibman et al. (2017)*,* Perica et al. (2022)*,* and Maldini et al. (2018)*

Combining metabolic engineering with pharmacological interventions can enhance CAR-T cell function under conditions of low antigen density and prolonged target cell engagement. Rapamycin has been shown to enhance the capacity of CAR-T cells to suppress HIV replication and delay viral rebound in vivo. These effects are likely attributable to rapamycin’s ability to preserve mitochondrial fitness and limit terminal differentiation in CAR-T cells [151] Current evidence suggests that engineering effective access to viral reservoirs requires consideration not only of antigen-binding properties but also of the intracellular signaling networks and metabolic fitness of CAR-T cells. Although substantial progress has been made, significant challenges remain, including viral latency, restricted tissue access, heterogeneous antigen expression, and immune-mediated suppression. Consequently, next-generation antiviral CAR-T cell strategies are being developed to enhance tissue trafficking, improve metabolic fitness, and optimize targeting of viral reservoirs rather than relying solely on cytotoxic activity [136, 152–155] (Fig. 4).

### Immune escape, selection pressure, and realistic therapeutic goals

One of the key unresolved questions is the extent to which CAR-T cell-mediated selective pressure promotes viral immune escape. CAR-T cells targeting a single viral epitope are particularly susceptible to the selection of escape variants and antigen downregulation, even when directed against conserved viral epitopes, particularly in genetically diverse viruses such as HIV. Clinical evidence from studies of bNAb-derived CAR-T cells demonstrates partial reductions in viral reservoir size but not durable virological control, suggesting insufficient antigen coverage or rapid viral adaptation [110]. The data indicate that CAR multiplexing, combination antigen targeting, and/or the use of broadly acting immune and /or pharmacological interventions may eliminate the issue of escape.

Emerging evidence suggests that achieving durable viral remission may represent a more realistic therapeutic objective than complete viral eradication. Accordingly, CAR-T cell therapy is likely to be most effective when combined with adjunctive therapies, including latency-reversing agents, metabolic reprogramming strategies, and immune checkpoint blockade, to promote sustained immune surveillance of viral reservoirs [139].

Overall, advances in antiviral CAR-T cell therapy reflect a transition from engineering strategies originally developed for oncology to the development of cell therapies tailored for chronic viral infections. Future progress will depend on integrating reservoir biology, virology, immune engineering, and advanced CAR-T cell manufacturing to enable durable viral control and, ultimately, achieve long-term remission.

## CAR-T and autoimmune disease: the rise of car-tregs and autoreactive car depletion

The application of CAR-T cell therapy to autoimmune diseases represents a paradigm shift from augmenting immune responses to restoring immune tolerance. Engineered cellular therapies are increasingly being developed to suppress pathogenic immune responses through antigen-specific CAR-regulatory T (CAR-Treg) cells or to selectively eliminate autoreactive immune cells using cytotoxic CAR-T cells, highlighting the broader role of CAR-T cells as programmable modulators of immune homeostasis beyond their established role as anticancer therapeutics [156].

### CAR-Tregs: active immune suppression with antigen specificity

Regulatory T (Treg) cells play a central role in maintaining immune tolerance and preventing autoimmunity. However, in autoimmune diseases, Treg cell number may be insufficient, or their suppressive function may be impaired. To address these limitations, CAR-Treg cells have been engineered and have shown promising efficacy in restoring antigen-specific immune tolerance and preventing disease progression in preclinical models, as well as in early-phase clinical studies of transplantation and autoimmune diseases [157–160].

In recent years, substantial advances in CAR design and engineering strategies have enabled the development of CAR-Treg therapy. CARs direct Treg suppressive activity to sites where their cognate antigen is expressed, thereby promoting localized immune regulation while minimizing systemic immunosuppression. These advances establish CAR-Tregs as an engineered platform for restoring antigen-specific immune tolerance [161]. Preclinical studies show that CAR-Tregs are able to deal with inflammation caused by inflammatory responses in autoimmune and transplant contexts without compromising the efficacy of the immune system [113, 162].

The development of CAR-Tregs remains challenging because of the need to maintain Treg lineage stability following genetic engineering and exposure to inflammatory microenvironments. CAR expression alone does not compromise the stability of human Tregs, provided that CAR signaling strength and co-stimulatory domain selection are carefully optimized to prevent excessive activation. Accordingly, CAR-Treg engineering must incorporate the principles of conventional CAR-T cell engineering while preserving Treg lineage stability and suppressive function [163].

Mechanistic studies have demonstrated that FOXP3 expression alone is insufficient to ensure stable Treg lineage identity and suppressive function. Establishing durable immune tolerance by Tregs requires maintaining the Treg-specific epigenetic program, including sustained demethylation of the FOXP3 T-cell-specific demethylated region (TSDR) and preserving an epigenetic landscape resistant to inflammatory reprogramming [164]. This concern is especially significant in autoimmune niches characterized by elevated levels of IL-6, IL-1β, and type I interferons, as these cytokines can drive Treg cell instability and facilitate conversion to an effector phenotype [158].

Although CAR-Tregs mediate antigen-specific immunosuppression, they remain challenged by heterogeneous tissue antigen expression, which may limit their therapeutic efficacy and promote excessive immunosuppression in tissues where protective immune responses must be preserved. Achieving an optimal balance between immune suppression and protective immunity therefore remains a major challenge [162].

Despite encouraging preclinical results, several key questions remain regarding the long-term durability, functional stability, and maintenance of engineered regulatory programs in CAR-Tregs. Addressing these challenges will be critical for the successful clinical translation of CAR-Treg therapy into late-stage clinical trials.

### Antigen-depleting CAR-T cells for autoimmunity

In addition to tolerogenic strategies that restore immune tolerance, cytotoxic CAR-T cells have emerged as a complementary therapeutic strategy for the selective elimination of pathogenic immune cells in autoimmune diseases. In disorders such as systemic lupus erythematosus and other antibody-mediated autoimmune diseases, autoreactive B cells and long-lived plasma cells are major drivers of disease pathogenesis. Selective elimination of these pathogenic cell populations represents a promising strategy for achieving durable disease control and preventing relapse [156, 165].

Early clinical studies of CD19-targeting CAR-T cells have demonstrated encouraging clinical outcomes, including the selective elimination of autoreactive B cells and durable drug-free remission, supporting the concept of immune resetting following CAR-T cell therapy. This therapeutic strategy can be extended to plasma cell depletion through the use of BCMA-targeting CAR-T cells, thereby expanding the therapeutic scope of CAR-T cell therapy in autoimmune diseases [166].

The long-term durability of immune resetting remains uncertain because autoreactive clones may re-emerge following immune reconstitution. Accordingly, establishing durable immune tolerance is likely essential for sustained remission, rather than relying solely on the elimination of pathogenic clones. Moreover, the adverse consequences of immunosuppression, including increased susceptibility to infections, hypogammaglobulinemia, and impaired vaccine responses, remain important safety concerns [167].

It is important to distinguish between immune-ablative CAR-T cell therapies and CAR-Treg-based strategies designed to restore antigen-specific immune tolerance. CAR-Tregs are designed to suppress antigen-specific immune responses, whereas cytotoxic CAR-T cells eliminate pathogenic immune cell populations. Future therapeutic strategies may integrate or sequentially deploy these approaches to induce immune resetting while reinforcing antigen-specific immune tolerance [168, 169].

### Immune reconstitution and tolerance re-establishment after CAR-T therapy

One of the primary goals of CAR-T cell therapy for autoimmune diseases is the establishment of durable immune tolerance through controlled immune reconstitution. Clinical evidence from CD19-targeting CAR-T cell therapy suggests that durable remission is likely mediated by the reconstitution of a more tolerant immune repertoire rather than complete eradication of all autoreactive clones, analogous to the immune resetting observed following hematopoietic stem cell transplantation [170–172]. The field of CAR-T cell engineering is evolving toward increasingly sophisticated therapeutic strategies, shifting its focus from simply enhancing cytotoxic activity to addressing the distinct biological features of different diseases. This emerging paradigm emphasizes precise antigen selection, disease-specific immune modulation, and long-term cellular persistence as the foundation of next-generation cellular therapies.

Recent clinical successes with CD19-targeting CAR-T cell therapy in autoimmune diseases provide compelling proof of principle for therapeutic immune reprogramming. Future research should focus on elucidating the mechanisms underlying loss of immune tolerance and disease recurrence, and on optimizing the integration of regulatory and cytotoxic CAR-T cell therapies to achieve durable immune homeostasis and sustained clinical remission [170–172].

## Safety, controllability, and manufacturability: translating molecular advances into patients

The clinical success of next-generation CAR-T cell therapies will depend on maximizing antitumor efficacy while integrating robust safety mechanisms, precise control of CAR-T cell activity, and scalable manufacturing platforms. Next-generation CAR-T cell platforms are advancing through innovations in molecular safety engineering and programmable control circuits, which, together with standardized manufacturing processes, are enabling the clinical translation of increasingly sophisticated receptor designs [11, 173].

### Built-in safety systems

Next-generation CAR-T cell therapies increasingly incorporate built-in safety mechanisms, such as the inducible caspase-9 (iCasp9) suicide switch. Administration of a small-molecule dimerized rapidly activates iCasp9, enabling the elimination of CAR-T cells within hours and thereby mitigating severe treatment-related toxicities [174].

In addition to irreversible suicide switches, reversible pharmacological control systems enable reversible modulation of CAR-T cell activity through small-molecule regulators, permitting dynamic management of treatment-related toxicities such as CRS and neurotoxicity while preserving therapeutically active CAR-T cell populations [175].

Logic-gated CAR-T cell systems provide an additional layer of safety by activating T cells only in the presence of predefined antigen combinations, thereby reducing off-tumor toxicity. SynNotch circuits further enhance target specificity through sequential antigen recognition, whereby recognition of a primary antigen induces CAR expression against a secondary antigen [114, 176, 177].

### Allogeneic “off-the-shelf” products and non-viral manufacturing

As programmable molecular control systems continue to evolve, considerable efforts are focused on overcoming the manufacturing limitations of autologous CAR-T cell therapies. The development of allogeneic, off-the-shelf CAR-T cell products has the potential to reduce manufacturing costs, shorten the time from patient identification to treatment, and expand product availability through standardized manufacturing processes. However, for these approaches to achieve broad clinical application, effective strategies are required to prevent graft-versus-host disease (GVHD) and evade host immune rejection [178].

The development of allogeneic CAR-T cell therapies relies on multiplex gene editing. CRISPR-Cas9-mediated deletion of endogenous T-cell receptor (TCR) chains and human leukocyte antigen (HLA) molecules enables the generation of universal donor-derived CAR-T cell products with substantially reduced alloreactivity. Clinical studies have demonstrated that these products can be manufactured at scale using these gene-editing strategies, supporting their broad clinical applicability. [179] Comprehensive analyses further highlight the immunological, logistical, and regulatory challenges that must be addressed before allogeneic platforms can supplant autologous therapy [180].

Manufacturing innovations are increasingly focused on achieving precise and reproducible CAR transgene integration. Approaches such as site-specific gene knock-in and non-viral delivery of CAR transgenes reduce variability in transgene expression while improving genomic integrity, manufacturing scalability, and product consistency compared with conventional viral vector-based approaches [181, 182].

The use of mRNA enables transient CAR expression in engineered cells, allowing reversible CAR activity without permanent genomic modification [183].

As the diversity of CAR-T cell products continues to expand, regulatory oversight and manufacturing standardization have become increasingly important. Allogeneic CAR-T cell products and non-viral manufacturing platforms must satisfy stringent standards for genomic integrity, manufacturing reproducibility, and minimal immunogenicity. Regulatory approval will require comprehensive characterization of genetically engineered products and their long-term safety profiles, particularly for multiplex-edited products and CAR-T cells incorporating complex synthetic gene circuits [184, 185].

**Fig. 5:**
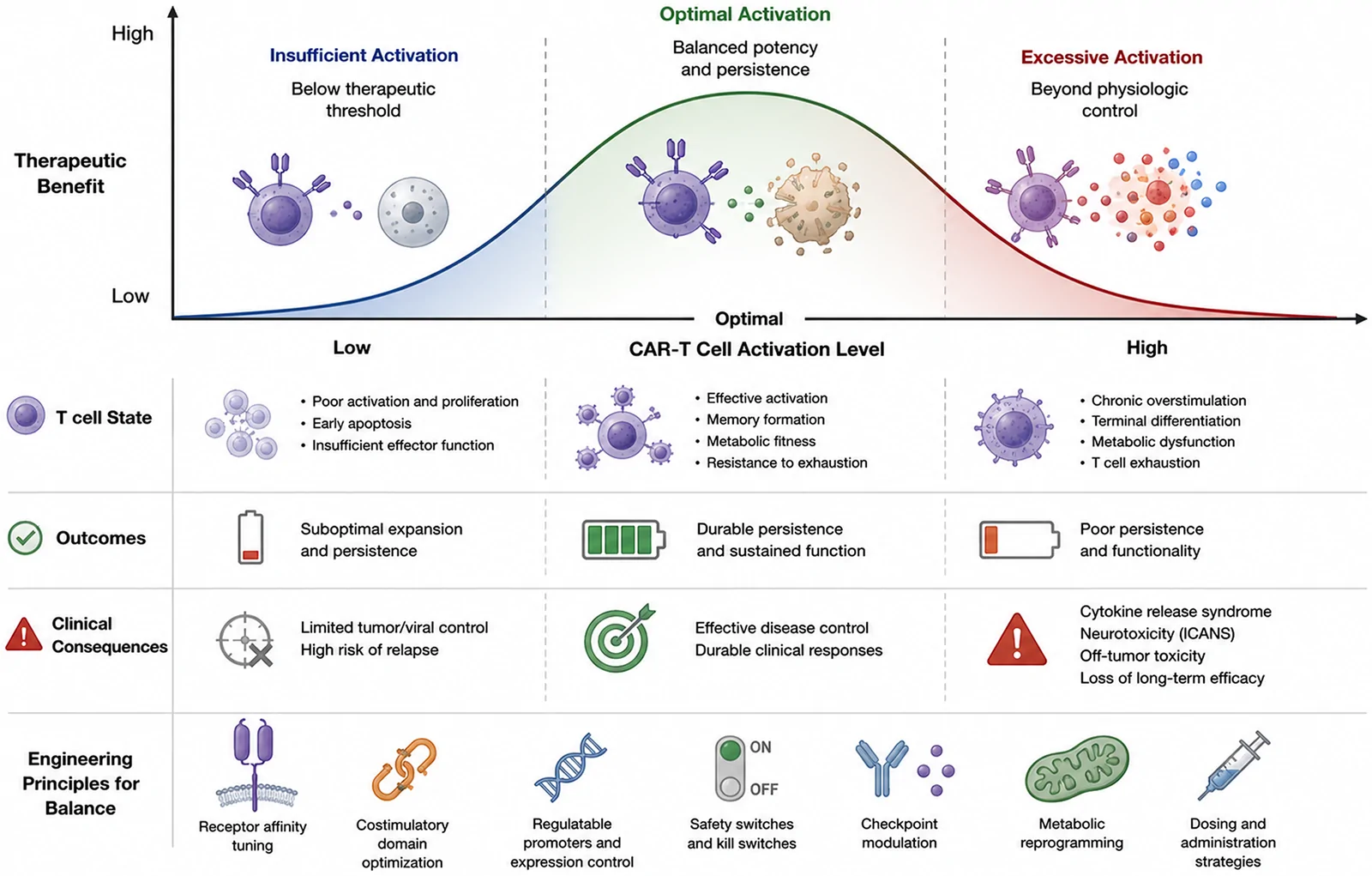
Balancing CAR-T-cell activation, persistence, and safety The therapeutic efficacy of CAR-T cell therapy depends on achieving an optimal balance among CAR-T cell activation, persistence, and physiological immune regulation. Insufficient CAR-T cell activation limits antitumor and antiviral efficacy, whereas excessive CAR signaling can lead to treatment-related toxicities, including CRS, ICANS, terminal T-cell differentiation, metabolic dysfunction, and T-cell exhaustion. Accordingly, next-generation engineering strategies seek to fine-tune CAR-T cell activation rather than maximize it, with the goal of achieving durable therapeutic responses while preserving long-term immune homeostasis. *This conceptual framework was developed based on previous studies describing CAR-T cell activation dynamics*,* immune-mediated toxicities*,* immune checkpoint regulation*,* T-cell exhaustion*,* and physiological immune homeostasis by Wherry and Kurachi (2015)*,* Blank et al. (2019)*,* Lim and June (2017)*,* June and Sadelain (2018)*,* and Ho and Kaech (2022)*

Advances in molecular safety engineering, programmable control systems, and scalable manufacturing technologies have transformed the clinical translation of CAR-T cell therapies. Whereas earlier efforts primarily focused on enhancing antitumor efficacy, current engineering strategies emphasize precise control of CAR-T cell activity, manufacturing reproducibility, and regulatory compliance as essential determinants of broad clinical implementation [13, 186] (Fig. 5) (Table 2).

**Table 2:** Preclinical evidence supporting molecular strategies to enhance CAR-T cell efficacy across disease contexts

Molecular mechanism	Disease context	Preclinical model(s)	Key mechanistic findings	Biological implications for CAR-T design	Representative references
Logic-gated and multi-antigen CAR signaling	Cancer	Human xenograft mouse models; in vitro mixed-antigen cultures	AND/OR gate CARs require combinatorial antigen recognition, reducing off-tumor activation while maintaining tumor killing	Antigen sensing can be engineered to reflect tissue-specific antigen logic rather than single-marker recognition	[17, 187]
Switchable / adaptor CAR platforms	Cancer; viral diseases	NSG mouse xenografts; human tumor cell lines	CAR activity is dose-dependent on adaptor presence, enabling reversible and titratable cytotoxicity	CAR-T activity can be pharmacologically controlled, improving safety and adaptability	[188, 189]
Affinity tuning of antigen-binding domains	Cancer; viral diseases	In vitro antigen-density gradients; murine tumor models	Lower-affinity CARs preferentially respond to high antigen density, sparing normal tissues	Activation thresholds can be programmed at the receptor level	[17, 111]
CRISPR-mediated TRAC knock-in of CARs	Cancer; universal platforms	Human T cells; murine leukemia models	Physiologic CAR expression reduces tonic signaling, delays exhaustion, and improves persistence.	The genomic context of CAR expression is a determinant of T-cell fate	[8]
Multiplex genome editing (checkpoint disruption)	Cancer; viral diseases	Human CAR-T cells; solid tumor xenografts	PD-1, LAG-3, or CTLA-4 deletion enhances effector function and tumor control	Inhibitory signaling pathways can be rewired intrinsically	[39, 190]
Dominant-negative cytokine receptors (e.g., dnTGF-βR)	Cancer	Murine solid tumor models	CAR-T cells become resistant to TGF-β–mediated suppression, maintaining proliferation and cytotoxicity	Suppressive cytokine signals can be blocked at the receptor level	[191]
Cytokine armoring (IL-12, IL-15, IL-18)	Cancer	Syngeneic and xenograft tumor models	Local cytokine secretion remodels the tumor microenvironment and recruits endogenous immunity	CAR-T cells can act as local immune orchestrators	[192, 193]
Transcription factor modulation (c-Jun overexpression)	Cancer	Mouse solid tumor models; in vitro chronic stimulation	Restores AP-1/NFAT balance, suppresses exhaustion gene programs	Exhaustion is an actively enforced transcriptional state that can be reprogrammed	[56],
TOX / NR4A pathway disruption	Cancer; viral diseases	Murine chronic infection and tumor models	Loss of TOX/NR4A partially restores effector function and proliferation	Exhaustion is genetically encoded and modifiable	[57, 194]
Epigenetic modulation during manufacturing	Cancer	Ex vivo CAR-T expansion with epigenetic modifiers	Preserves stem-like chromatin accessibility and memory-associated enhancers	Early epigenetic imprinting determines long-term CAR-T fate	[78, 195]
Metabolic reprogramming (PGC-1α, mitochondrial fitness)	Cancer; viral diseases	Murine tumors; chronic stimulation assays	Enhanced oxidative metabolism improves persistence and stress resistance	Metabolism is instructive, not merely supportive, for CAR-T function	[74, 124]
Nutrient transporter engineering (e.g., GLUT1)	Cancer	Murine tumor models	Improved glucose uptake enhances proliferation and effector capacity	Nutrient acquisition pathways can be engineered to overcome hostile niches	[196]
Latency-aware CAR designs (low antigen sensitivity)	Viral diseases (HIV, HBV)	Humanized mouse models; in vitro latency systems	CARs can recognize infected cells with sparse antigen expression	CAR sensitivity must be matched to viral reservoir biology	[108, 154]
Resistance to activation-induced cell death	Viral diseases	Chronic stimulation models	Enhanced survival under repeated antigen exposure	Longevity mechanisms are critical for chronic infections	[197]
CAR-Tregs with tuned signaling domains	Autoimmune diseases	Murine autoimmunity and transplant models	Antigen-specific CAR-Tregs suppress inflammation without systemic immunosuppression	CAR signaling strength determines regulatory vs. effector fate	[198, 199]
B cell–depleting CAR-T cells (CD19, BCMA)	Autoimmune diseases	Lupus-prone mouse models	Elimination of autoreactive B cells resets immune repertoire	CAR-T can induce immune “reset” rather than sustained activation	[200, 201]
Epigenetic stabilization of Treg identity	Autoimmune diseases	Inflammatory cytokine exposure models	Stable FOXP3-associated chromatin resists inflammatory reprogramming	Regulatory CAR-T therapies require lineage stabilization	[202]

## Clinical translation — where we are and what to watch for

**Fig. 6:**
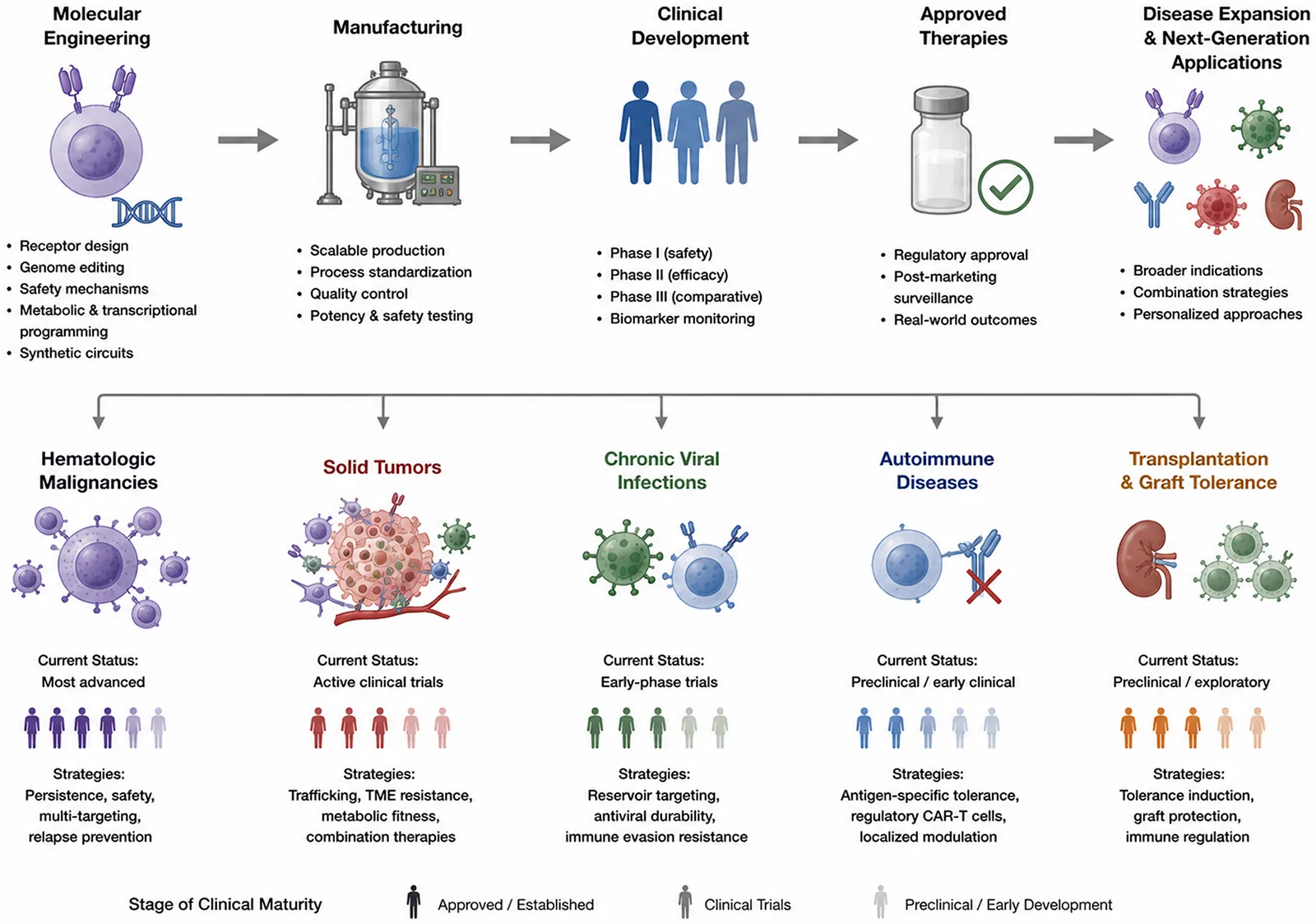
Clinical translation of next-generation CAR-T-cell engineering. Advances in molecular engineering have entered clinical practice in selected disease settings. Among current applications, hematological malignancies have achieved the greatest clinical success. In contrast, treating solid tumors, chronic viral infections, autoimmune diseases, and transplantation requires increasingly sophisticated engineering strategies to overcome distinct disease-specific barriers. To achieve broad clinical applicability, CAR engineering, genome engineering, molecular safety engineering, metabolic reprogramming, and scalable, standardized manufacturing must be integrated into unified therapeutic platforms. *This conceptual framework was developed based on previous studies describing the clinical translation of CAR-T cell therapies*,* CAR-T cell manufacturing platforms*,* regulatory science*,* and next-generation cellular engineering by June and Sadelain (2018)*,* Depil et al. (2020)*,* Rivière and Sadelain (2017)*,* Levine et al. (2017)*,* and Maus and June (2016)*

The clinical success of next-generation CAR-T cell therapies will depend on maximizing antitumor efficacy while integrating robust safety mechanisms, precise control of CAR-T cell activity, and scalable manufacturing platforms. Next-generation CAR-T cell platforms are advancing through innovations in molecular safety engineering and programmable control systems, which, together with standardized manufacturing processes, are enabling the development and clinical translation of increasingly sophisticated receptor designs [13, 203] (Fig. 6) (Table 3).

**Table 3:** Clinical trials of CAR-T cell therapy across cancer, viral diseases, and autoimmune diseases

Disease context	CAR-T target / strategy	Trial phase & population	Key clinical observations	Mechanistic insights	Trial identifiers (NCT)
Hematologic cancers	CD19 CAR-T (CD28 or 4-1BB costimulation)	Phase I–III; relapsed/refractory B-ALL, DLBCL	High CR rates; long-term remission in subsets	Persistence and memory differentiation correlate with durability; costimulatory domain shapes fate.	NCT02348216 (ZUMA-1); NCT02445248 (JULIET); NCT02435849 (ELIANA)
	BCMA CAR-T	Phase I–III; relapsed/refractory multiple myeloma	Deep responses: relapse associated with antigen loss or limited persistence	Antigen density and exhaustion constrain durability	NCT03361748 (KarMMa); NCT03548207 (CARTITUDE-1)
Solid cancers	HER2, EGFR, GD2, mesothelin CAR-T	Phase I–II; advanced solid tumors	Limited efficacy; manageable on-target toxicity	Poor trafficking and suppressive TME dominate	NCT00902044; NCT01837602; NCT02107963
	Armored CAR-T (cytokine-secreting / checkpoint-modulated)	Phase I; Refractory solid tumors	Enhanced local immune activation; increased inflammatory toxicity	Local immune modulation can overcome TME constraints	NCT02498912
Viral diseases (HIV)	CD4-ζ CAR-T	Phase I; ART-treated individuals	Long-term persistence; minimal reservoir impact	Antigen scarcity and latency limit efficacy	NCT01013415
	bnAb-derived CAR-T	Phase I–II; people living with HIV on ART	Safety demonstrated; modest effects on viral reservoirs	High-affinity recognition insufficient without latency reversal	NCT03617198
	Gene-edited CAR-T (CCR5 disruption)	Phase I; ART-suppressed individuals	Resistance to HIV infection; variable persistence	Intrinsic resistance pathways can be engineered	NCT03164135
Viral diseases (HBV)	HBV envelope–specific CAR-T	Phase I; chronic HBV infection	Transient viral suppression; hepatotoxicity risk	Tunable signaling is required for liver safety	NCT03899415
Autoimmune diseases (B cell–mediated)	CD19 CAR-T (B cell depletion)	Phase I–II; SLE, myasthenia gravis	Durable, drug-free remission in small cohorts	Immune repertoire “reset” via deep B-cell ablation	NCT03030976; NCT04146051
Autoimmune diseases (regulatory)	CAR-Tregs (HLA or tissue antigen–specific)	Phase I; transplantation, autoimmunity	Safety and persistence demonstrated	Antigen-specific suppression without global immunosuppression	NCT04817774
Autoantibody-mediated disease	CAAR-T (autoantigen bait CAR)	Phase I; pemphigus vulgaris	Selective elimination of autoreactive B cells	Precision deletion of pathogenic clones	NCT04422912

## Hematologic malignancies: continued optimization of persistence and safety is expanding durable remissions

Initially developed for the treatment of hematological malignancies, CAR-T cell therapy has evolved into a versatile cellular engineering platform capable of addressing solid tumors, chronic viral infections, autoimmune diseases, and transplantation. Although each disease presents distinct biological challenges, a common objective remains translating advances in receptor engineering, signaling regulation, and metabolic programming into safe and effective therapies. The following sections highlight how the engineering strategies discussed in this Review are adapted to address disease-specific mechanisms of immune escape, cellular persistence, and tissue-specific immune regulation across diverse disease contexts.

### Hematologic malignancies: optimizing persistence and safety to expand durable remissions

CAR-T cell therapy has revolutionized the treatment of hematological malignancies, enabling patients with relapsed or refractory acute lymphoblastic leukemia (ALL), diffuse large B-cell lymphoma (DLBCL), and multiple myeloma to achieve durable remissions. The broad spectrum of clinical outcomes observed following CAR-T cell therapy reflects its evolution from proof of concept to a mature therapeutic platform characterized by improved antitumor efficacy, long-term durability, sophisticated immune modulation, and expanding clinical applicability [204].

Durable clinical responses depend on the successful generation of CAR-T cells that maintain a memory-like rather than an exhausted T-cell phenotype. The durability of CAR-T cell responses depends on multiple factors, including CAR construct design, the choice of co-stimulatory domain, and host immune factors [126, 204, 205].

The long-term efficacy of CAR-T cell therapy must be balanced against treatment-related immune toxicities, particularly CRS and immune effector cell-associated neurotoxicity syndrome (ICANS). Current clinical management focuses on patient selection, dose optimization, and cytokine-directed interventions. Meanwhile, novel engineering strategies are being developed to precisely modulate CAR-T cell activity without compromising antitumor efficacy [204, 206].

Pharmacological interventions following CAR-T cell infusion provide an additional layer of control over CAR-T cell activity. Selective kinase inhibitors can enhance antitumor efficacy and attenuate T-cell exhaustion by modulating CAR-T cell signaling without compromising cytotoxic function, while preserving the benefits of genetically engineered CAR-T cell products [207, 208].

A major challenge in CAR-T cell therapy is elucidating the mechanisms underlying tumor immune escape and the longitudinal fate of CAR-T cells after infusion. Disease relapse may result from antigen loss, lineage switching, or CAR-T cell dysfunction. Longitudinal monitoring of CAR-T cell persistence, functional status, and immune reconstitution may facilitate early prediction of disease relapse and inform consolidation strategies, such as dual-target CAR-T cell therapy, sequential CAR-T cell infusion, or rational combination therapies [1, 204, 209].

Collectively, these advances indicate that CAR-T cell therapy for hematological malignancies has entered a new phase of clinical translation. However, durable remissions across diverse malignancies and patient populations remain inconsistent, underscoring the need for a deeper mechanistic understanding of tumor immunology, optimized manufacturing processes, and rigorous clinical evaluation to achieve durable efficacy and long-term safety.

### Solid tumors: re-engineering CAR-T cell signaling, metabolism, and tissue navigation to overcome stromal immunosuppression and antigenic heterogeneity

Despite the remarkable clinical efficacy of CAR-T cell therapy in hematological malignancies, its application to solid tumors remains limited by the complex and immunosuppressive tumor microenvironment (TME). Solid tumors present multiple structural, biochemical, and metabolic barriers that restrict CAR-T cell infiltration, persistence, and effector function. Consequently, successful CAR-T cell therapy for solid tumors requires not only precise tumor targeting but also remodeling of the local tumor immune microenvironment [210–212]. Solid tumors present multiple interrelated barriers, including impaired CAR-T cell trafficking, stromal barriers, metabolic constraints, and chronic immunosuppression, that collectively limit CAR-T cell persistence and antitumor efficacy even after antigen recognition [210, 213].

Genetic engineering strategies that enhance CAR-T cell function by remodeling the local tumor immune microenvironment are increasingly being incorporated into next-generation CAR-T cell platforms [211, 212]. One of the most promising engineering strategies involves expressing a dominant-negative transforming growth factor-β receptor II (dnTGF-βRII), which blocks TGF-β-mediated immunosuppressive signaling and preserves CAR-T cell effector function [70, 119, 214]. Preclinical studies have demonstrated that TGF-β pathway blockade enhances CAR-T cell proliferation and antitumor efficacy across multiple solid tumor models, whereas early-phase clinical trials in PSMA-positive prostate cancer have demonstrated preliminary biological activity but have also reported significant treatment-related toxicities [214–216].

Cytokine armoring illustrates the balance between enhanced antitumor efficacy and treatment-related toxicity. This is exemplified by early T cells Redirected for Universal Cytokine Killing (TRUCKs), which secreted cytokines in response to CAR-mediated antigen recognition, thereby remodeling the tumor microenvironment [217, 218]. Preclinical studies have demonstrated that IL-15 promotes CAR-T cell persistence, memory-like differentiation, enhanced mitochondrial fitness, and sustained antitumor activity under conditions of chronic antigen stimulation [219, 220]. However, clinical studies have shown that IL-15 overexpression may increase the risk of severe CRS, necessitating the integration of built-in safety mechanisms, such as the inducible caspase-9 (iCasp9) suicide switch, which enables rapid elimination of CAR-T cells in response to severe treatment-related toxicities [221, 222].

Strategies that remodel the tumor microenvironment (TME) through the localized delivery of interleukin-12 (IL-12) or interleukin-18 (IL-18) enhance dendritic cell maturation, promote macrophage polarization, and facilitate T-cell recruitment. To minimize systemic toxicity while achieving localized immune activation through tumor-restricted cytokine delivery, several engineering strategies have been developed, including membrane-tethered cytokine expression, NFAT-responsive promoters, and synthetic Notch (synNotch) circuits [223–225]. Collectively, these strategies reflect an improved understanding of the requirements for effective antitumor immune responses in solid tumors during CAR-T cell therapy. Rather than relying solely on the cytotoxic activity of CAR-T cells, successful treatment depends on coordinated interactions among multiple immune and stromal cell populations within the tumor microenvironment.

Furthermore, metabolic engineering further extends CAR-T cell armoring strategies by preserving mitochondrial fitness under conditions of nutrient deprivation within the tumor microenvironment [226, 227].

Ultimately, one of the greatest barriers to durable clinical responses in solid tumors is intratumoral antigen heterogeneity. Unlike CD19-positive hematological malignancies, solid tumors exhibit spatial and temporal heterogeneity in antigen expression, enabling immune escape and tumor recurrence despite CAR-T cell therapy [228]. Armored CAR-T cells that activate endogenous immune responses via cytokine secretion or checkpoint blockade can increase epitope spreading and bystander killing [229, 230]. However, achieving durable tumor control will require an integrated therapeutic strategy that combines multi-antigen targeting, coordinated immune modulation, and adaptive regulatory circuits capable of adapting to tumor evolution.

Collectively, these advances have transformed armored CAR-T cells from optimized cytotoxic effectors into programmable immune modulators capable of precisely modulating tissue-specific immune responses. Future efforts should focus on establishing design principles for next-generation CAR-T cell products that integrate engineering sophistication, safety, manufacturability, and scalability to support broad clinical translation.

### Viral diseases: CAR-T for HIV, HBV, and other persistent infections

Chronic viral infections, such as HIV and HBV, evade immune-mediated clearance through the establishment of persistent viral reservoirs, necessitating lifelong antiviral therapy. To address this challenge, CAR-T cell therapy has emerged as a promising therapeutic strategy. Accordingly, current engineering strategies have evolved beyond targeting viral infection alone to focus on eliminating viral reservoirs, modulating antiviral immune responses, and enhancing CAR-T cell persistence [139, 231].

#### CAR-T cell therapy for HIV infection

HIV is one of the most extensively investigated targets for CAR-T cell therapy. The major challenge in achieving an HIV cure lies in identifying latently infected cells that express little or no viral antigen, together with overcoming viral escape mutations, T-cell dysfunction, and limited immune access to viral reservoirs. These challenges have shifted the therapeutic focus toward sustained immunological surveillance capable of eliminating reactivated infected cells before viral rebound occurs [232–234].

Compared with conventional cytotoxic T lymphocytes (CTLs), CAR-T cell therapy offers several advantages, as CAR-T cells recognize HIV-infected cells independently of major histocompatibility complex (MHC)-restricted antigen presentation, thereby overcoming a key mechanism of viral immune evasion. Early-generation CD4-based CAR-T cells demonstrated the feasibility and safety of this approach but failed to achieve durable control of HIV infection, highlighting the need for further optimization of CAR design [235, 236]. Advances in CAR-T cell engineering for cancer have informed the development of HIV-targeted CAR constructs. Second-generation CARs incorporating CD28 or 4-1BB costimulatory domains enhance CAR-T cell activation and persistence, with CD28 preferentially promoting effector T-cell differentiation, whereas 4-1BB supports memory-like differentiation and improves metabolic fitness [237, 238]. This modified CAR exhibits higher antiviral activity in vivo and better control of HIV infection in humans [239]. In addition to CD4-based CARs, broadly neutralizing antibody (bNAb)-based CARs target conserved epitopes within the HIV envelope glycoprotein, thereby broadening antiviral coverage. Bispecific CARs incorporating both CD4 and bNAb domains enhance targeting specificity and reduce viral escape, although off-target effects remain a challenge [118, 239, 240]. Overall, these advances contributed to enhanced efficacy and sustainability of CAR-T therapy for HIV [241, 242].

#### Persistence, resistance, and reservoir targeting

Durable CAR-T cell persistence is critical for effective HIV therapy, as antiretroviral therapy suppresses viral antigen levels, thereby limiting antigen-driven CAR-T cell expansion. To overcome this limitation, complementary strategies, including therapeutic vaccination and repeated CAR-T cell infusions, may help sustain functional CAR-T cell populations. Vaccine-boosted CAR-T cell therapy has shown encouraging results in preclinical cancer models and may represent a promising strategy for HIV treatment [243–245].

HIV can directly infect CAR-T cells, necessitating strategies to render these cells resistant to viral infection. CCR5-targeted gene-editing strategies, including site-specific gene knock-in approaches that simultaneously integrate the CAR transgene and disrupt CCR5, enhance resistance to HIV infection. Additional approaches, including CCR5-targeted siRNAs and viral fusion inhibitors, have also been investigated. Collectively, protecting CAR-T cells from HIV infection is essential for improving their persistence and therapeutic efficacy [205, 239, 246].

To overcome the limitations associated with the limited persistence of peripheral CAR-T cells, recent strategies have explored hematopoietic stem and progenitor cell (HSPC)-based CAR therapies. Genetically engineered HSPCs expressing CARs may provide durable engraftment, self-renewal, and sustained generation of antiviral effector cells [247, 248]. Studies in mouse and nonhuman primate models have demonstrated sustained generation of CAR-expressing immune cells and partial control of viral rebound following treatment interruption [248, 249]. Although the reservoirs could not be eradicated, these studies demonstrate that HSPC-based CAR therapies can provide durable immune surveillance.

#### Purging viral reservoirs and immune-privileged sites

The persistence of latent HIV reservoirs remains the principal barrier to achieving a functional cure. Although latency-reversing agents have demonstrated limited efficacy, they may enhance therapeutic efficacy when combined with CAR-T cell therapy. Engineering CAR-T cells to express CXCR5 enables targeted elimination of virus-infected T follicular helper cells residing within B-cell follicles and may further enhance antiviral efficacy when combined with immunomodulatory agents such as ALT-803 [250, 251].

#### Extension to HBV and other persistent infections

Although HIV has been the primary focus of antiviral CAR-T cell research, the principles established through these studies have guided the development of CAR-T cell therapies for other chronic viral infections. Similar to HIV, HBV persistence is driven by stable viral reservoirs, including covalently closed circular DNA (cDNA), together with T-cell exhaustion. Preclinical and early clinical studies have highlighted both the therapeutic potential and the risk of off-target hepatotoxicity associated with CAR-T cells targeting HBV surface antigens. Lessons learned from HIV regarding antigen specificity, immune evasion, and safety are expected to inform the development of CAR-T cell therapies for HBV and other chronic viral infections [252, 253].

CAR-T cell therapy for viral diseases has evolved from an experimental concept into a rapidly advancing therapeutic platform, building on decades of advances in cancer immunotherapy. Early clinical studies have demonstrated a favorable safety profile and provided evidence of antiviral activity, although its capacity to eradicate persistent viral reservoirs remains unproven. Further clinical translation will depend on integrating next-generation CAR designs with strategies that enhance CAR-T cell persistence in vivo, confer resistance to viral infection, and overcome viral reservoirs and anatomical barriers that limit immune surveillance. As these strategies continue to mature, CAR-T cell therapy is expected to become an integral component of combination therapeutic strategies aimed at achieving functional cures for HIV, HBV, and other chronic viral infections.

### Autoimmunity and transplantation: CAR-Tregs and targeted depletion strategies

The engineering strategies discussed in this review have demonstrated encouraging results in preclinical studies, but only a limited number have undergone rigorous clinical evaluation. Several emerging approaches, including synthetic gene circuits, advanced epigenetic programming, and metabolic engineering, remain at an early stage of development. Consequently, it remains unclear whether these approaches will translate into improved clinical efficacy, enhanced safety, more durable responses, and more cost-effective therapies. Further clinical studies are required to determine whether these strategies provide meaningful clinical benefit over current CAR-T cell therapies [254, 255]. (Table 4).

**Table 4:** Clinical Development Status of Major CAR-T Engineering Strategies

Technology	Representative Examples	Clinical Status	Evidence Level	Major Remaining Challenges
Second-generation CARs (CD28, 4-1BB)	CD19, BCMA CAR-T	Approved	Established clinical benefit	Relapse, toxicity, cost
Dual-target CARs	CD19/CD22, BCMA/GPRC5D	Clinical trials	Early clinical evidence	Antigen escape, complexity
Logic-gated CARs	SynNotch, AND-gate CARs	Early clinical trials	Limited clinical evidence	Manufacturing complexity
Armored CAR-T cells	IL-12, IL-15, IL-18 secreting CARs	Early clinical trials	Preliminary clinical evidence	Cytokine-related toxicity
Checkpoint-resistant CAR-T cells	PD-1 knockout, dominant-negative receptors	Early clinical trials	Preliminary clinical evidence	Long-term safety
CRISPR-edited CAR-T cells	TRAC knockout, PDCD1 knockout	Clinical trials	Early clinical evidence	Genomic safety
Multiplex genome-edited CAR-T cells	Universal allogeneic CAR-T products	Early clinical trials	Limited clinical evidence	Translocations, persistence
Epigenetic reprogramming	c-Jun overexpression, chromatin remodeling	Preclinical	Proof-of-concept	Clinical validation
Metabolic engineering	PGC-1α, mitochondrial optimization	Preclinical	Proof-of-concept	Durability, safety
Synthetic gene circuits	SynNotch, inducible CARs	Preclinical/Early clinical	Limited clinical evidence	Regulatory complexity
CAR-T for HIV/HBV	HIV-directed CAR-T cells	Early clinical trials	Proof-of-concept	Reservoir eradication
CAR-Tregs	Autoimmunity/transplantation	Early clinical trials	Preliminary evidence	Stability and persistence

Autoimmune diseases and solid-organ transplantation are examples in which a healthy immune system does not function at its full potential; rather than an immune system that is too weak (due to cancer), both reflect an immune system that is too active or misdirected. While the use of drugs to inhibit the activity of the immune system to help to prevent rejection of transplanted organs is one method used to avoid organ rejection from occurring, this method has significant drawbacks, with an increased incidence of serious side effects from medications such as cardiovascular disease and the potential for developing new cancers and infection with opportunistic pathogens, to name a few [256]. In autoimmune diseases, immunosuppressive medications are often effective in controlling symptoms; however, they are associated with suboptimal long-term immune function. As a result, there has been significant interest in using cells as therapies to repair and modulate the immune system with greater specificity. Among cellular therapy options, Tregs (regulatory T cells) have attracted increasing attention, particularly CAR-Tregs, which target specific antigens [160, 257].

#### Rationale for CAR-Tregs in transplantation and autoimmunity

Transplant tolerance, defined as long-term graft acceptance in the absence of lifelong immunosuppressive therapy, has long been a central goal of transplantation medicine. Observations of spontaneous tolerance in liver and selected kidney transplant recipients, together with findings from preclinical studies, have identified regulatory T cells (Tregs) as key mediators of transplant tolerance. These findings have driven the development of adoptive Treg therapy [187, 258, 259].

Early research employing polyclonally expanded Tregs in kidney transplant recipients provided evidence of safety and an empirical observation of decreased immunosuppression in certain patients [116, 260–262]. A major limitation of polyclonal Treg therapy is its lack of antigen specificity, necessitating the infusion of large numbers of cells to achieve therapeutic efficacy. In contrast, studies have demonstrated that alloantigen-specific Tregs exhibit superior immunosuppressive activity in both in vitro and in vivo models, enabling effective control of allograft immune responses at substantially lower cell doses [263, 264]. Building on these findings, researchers have engineered Tregs to selectively recognize donor-specific antigens, particularly mismatched HLA molecules, through CAR technology.

#### CAR-Treg engineering and functional principles

CAR-Tregs share a structural architecture similar to that of conventional CAR-T cells but are designed to induce antigen-specific immune tolerance rather than mediate cytotoxicity. Most CAR-Tregs employ second-generation CAR constructs that provide the signaling required for Treg activation and function by incorporating CD3ζ together with a costimulatory domain, such as CD28 or 4-1BB [265].

The choice of costimulatory domain is a critical determinant of CAR-Treg function. Unlike conventional CAR-T cells, CD28 more effectively preserves Treg stability, lineage-defining gene expression, and suppressive function, whereas 4-1BB, although promoting persistence, may compromise CAR-Treg stability and suppressive activity. These findings underscore the need to optimize CAR-Treg constructs specifically for immune regulation rather than directly adapting CAR-T cell designs developed for cancer immunotherapy [266, 267].

#### Antigen selection and HLA-specific CAR-Tregs

Solid organ transplantation represents an ideal clinical setting for the application of CAR-Tregs targeting mismatched donor HLA molecules selectively expressed within the transplanted allograft. Among these targets, HLA-A2 has been the most extensively investigated owing to its high prevalence in the general population [268]. Multiple preclinical studies have demonstrated that HLA-A2-specific CAR-Tregs exhibit selective localization, preferentially accumulate within HLA-A2-positive allografts, and provide superior protection against allograft rejection compared with polyclonal Tregs in humanized mouse models [113, 269].

CAR-Tregs can incorporate scFvs derived from either murine or human antibodies. The origin and composition of the scFv are critical determinants of antigen specificity, safety, and immunogenicity. Because murine-derived scFvs may elicit immune responses in human recipients, current engineering strategies increasingly focus on developing humanized scFvs or scFvs derived from fully human monoclonal antibodies [270, 271]. Comprehensive characterization of HLA epitope specificity is also essential. Because individual HLA epitopes may be shared across multiple HLA alleles, there is a potential risk of off-target cross-reactivity [272]. Conversely, identifying shared HLA epitopes may broaden the applicability of individual CAR-Treg products by expanding the range of HLA-mismatched transplant recipients that can be targeted by a single CAR specificity.

Beyond HLA-A2, expanding the repertoire of CAR-Tregs targeting additional HLA epitopes will be a critical priority. Given the geographic variation in HLA allele frequencies, a modular panel of CAR-Treg products tailored to regional HLA distributions will likely be required to maximize their clinical applicability in transplantation.

#### Migration, local suppression, and mechanisms of action

The efficacy of CAR-Tregs in transplantation depends on their ability to selectively traffic to antigen-expressing allografts, where they mediate localized immunosuppression with greater efficacy than polyclonal Tregs. However, their limited capacity to suppress pre-existing memory T cells may reduce therapeutic efficacy in sensitized transplant recipients. Consequently, CAR-Tregs may be most effective in no sensitized recipients or when administered in the peri-transplant period [113, 188, 189, 269].

#### Persistence and cytokine dependence

To eliminate or substantially reduce the need for long-term pharmacological immunosuppression, CAR-Tregs must persist long term. Preclinical studies have demonstrated that CAR-Treg persistence within antigen-positive allografts exceeds that of polyclonal Tregs and ranges from several days to several weeks. However, these studies were performed in murine models, which do not fully recapitulate the human cytokine milieu required to support long-term Treg persistence and function [269, 273].

Cytokines such as IL-2, TGF-β, IL-7, and IL-33 play essential roles in supporting Treg proliferation, survival, and tissue residency. Although CAR signaling can transiently reduce the dependence on exogenous IL-2, long-term CAR-Treg persistence and function remain dependent on IL-2 and other supportive cytokine signals. Accordingly, low-dose IL-2 administration in combination with CAR-Treg therapy may enhance CAR-Treg persistence and therapeutic efficacy [274, 275]. While not yet thoroughly tested in Tregs, the use of fourth-generation CARs that deliver supportive cytokines (e.g., IL-2) to Tregs represents a promising opportunity [276].

#### Safety considerations: stability, tonic signaling, and control mechanisms

Safety is a critical consideration in CAR-Treg therapy, particularly with respect to the stable maintenance of FOXP3 expression and the prevention of conversion into effector T cells. Preclinical studies have demonstrated that CAR-Tregs maintain a stable regulatory phenotype. Accordingly, thymus-derived naïve Tregs are considered the preferred cell source for CAR engineering [194, 277, 278]. Given the influence of antigen-independent tonic signaling on CAR-Treg function, CAR design parameters require careful optimization [202, 278]. The incorporation of safety switches, such as inducible caspase-9 (iCasp9), truncated EGFR (EGFRt), or RQR8, enhances the safety profile of CAR-Treg therapy by enabling the selective elimination of CAR-Tregs when clinically indicated [174, 279–281].

#### Emerging clinical translation and future outlook

Although CAR-Tregs remain largely in the preclinical stage, early clinical translation is now underway. Preclinical evidence suggests that CAR-Tregs can mediate antigen-specific, graft-targeted immune regulation while reducing the need for systemic immunosuppression and its associated adverse effects. Nevertheless, several critical challenges remain, including the identification of optimal antigen targets, the long-term stability and persistence of CAR-Tregs, their capacity to maintain durable immune tolerance, and the need to balance effective immune regulation with the potential risks of infection and malignancy.

Combination strategies integrating CAR-Tregs with transient immunosuppressive therapy, low-dose cytokine therapy, or complementary regulatory cell therapies may provide the most effective strategy for achieving operational tolerance following transplantation. By integrating advances from oncology, autoimmune disease, and transplantation immunology, CAR-Treg therapy has the potential to emerge as a transformative therapeutic platform for restoring immune homeostasis by shifting from broad immunosuppression to antigen-specific immune regulation.

Early clinical studies have yielded encouraging results, suggesting that CAR-Tregs and B cell-depleting CAR-T cell therapies can induce more durable and antigen-specific immune tolerance than conventional immunosuppressive therapies. The successful clinical implementation of these therapies for autoimmune diseases and solid organ transplantation will depend on improving the efficacy and durability of CAR-T cell therapies, optimizing antigen selection, and ensuring long-term safety [282–284].

## Remaining challenges and future directions

CAR-T cell therapy has transformed the treatment of a broad range of diseases; however, the development of durable, effective, and broadly applicable cellular immunotherapies remains an ongoing challenge. Future advances are unlikely to be driven solely by iterative improvements in CAR architecture but rather by integrating advances in synthetic biology, genome engineering, immunometabolism, manufacturing, and regulatory science into integrated therapeutic platforms capable of adapting to diverse disease contexts.

### Durable reversal of epigenetically fixed exhaustion

One of the major challenges facing next-generation CAR-T cell therapy is reversing the transcriptional and epigenetic programs that drive T-cell exhaustion. Although transient interventions can partially restore T-cell function, achieving durable functional reprogramming will likely require targeted epigenetic editing strategies and modulation of exhaustion-associated transcription factors, such as TOX and NR4A, while preserving stem-like memory T-cell populations and ensuring genomic integrity [285, 286].

### Antigen escape and target heterogeneity

Antigen loss remains a major mechanism of therapeutic resistance in CAR-T cell therapy, together with intratumoral antigen heterogeneity, particularly in solid tumors and highly mutated malignancies. Selective pressure imposed by CAR-T cells promotes the emergence of antigen-negative tumor clones, thereby facilitating immune escape and disease relapse [8, 246]. Consequently, multispecific CAR constructs, combinatorial CAR designs, logic-gated CARs, and adaptor-based targeting systems have been developed to overcome the limitations of single-antigen targeting and reduce the risk of antigen escape [277, 287]. However, the development of these strategies does not fully address the dynamic antigen evolution of these phenotypes.

### Context-specific safety and localized immunomodulation

As CAR-T cell therapies become increasingly potent, maintaining an optimal balance between efficacy and safety has become a central challenge. Systemic CAR-T cell activation can lead to severe treatment-related toxicities, including CRS, ICANS, and on-target, off-tumor toxicity, particularly when the target antigen is also expressed at low levels in normal tissues [288, 289]. Accordingly, increasing emphasis has been placed on engineering strategies that provide spatially and temporally controlled regulation of CAR-T cell activity. These approaches include inducible CAR expression, synNotch-based logic circuits, and CAR-T cells engineered to secrete cytokines or immune checkpoint inhibitors selectively within diseased tissues, thereby maximizing on-target efficacy while minimizing systemic toxicity [279, 280]. Preclinical studies have demonstrated that localized delivery of immunomodulatory agents, including IL-12, IL-15, and immune checkpoint-blocking antibodies, enhances antitumor efficacy without significantly increasing toxicity compared with systemic administration [281, 290]. The development of context-responsive CAR-T cell systems will be critical for the future clinical translation of CAR-T cell therapies for autoimmune diseases, transplantation, and chronic viral infections, where preserving immune tolerance while maintaining therapeutic efficacy is essential.

### Manufacturing standardization and scalability

Manufacturing remains a major challenge for the widespread clinical implementation of CAR-T cell therapies. Autologous CAR-T cell products are time-consuming and costly to manufacture and exhibit variable product quality owing to differences in patient-derived T cells. Allogeneic CAR-T cell therapies offer a promising alternative but require extensive genome engineering to prevent host immune rejection and graft-versus-host disease (GVHD). Continued advances in closed-system manufacturing, standardized cell sources, and genome engineering are expected to improve the scalability, manufacturing reproducibility, and broad clinical applicability of CAR-T cell therapies [291, 292]. Despite substantial advances in CAR-T cell therapy, several important challenges remain. CAR-T cell therapy continues to rely on complex, patient-specific manufacturing processes that are time-consuming and costly and contribute to variability in product quality and clinical outcomes. Although allogeneic platforms and nonviral manufacturing approaches may improve scalability, challenges related to manufacturing standardization, supply chain logistics, and regulatory approval remain. Consequently, broader clinical implementation will depend on continued advances in manufacturing technologies and cellular engineering strategies [9, 57, 293–295].

### Regulatory pathways and long-term surveillance

As CAR-T cell therapies incorporate increasingly sophisticated genome engineering, synthetic biology, and autonomous regulatory circuits, they also introduce additional safety and regulatory challenges, including genomic instability, insertional mutagenesis, immunogenicity, and delayed toxicities. Accordingly, long-term patient follow-up, post-marketing surveillance, and evolving regulatory frameworks will be essential to ensure patient safety while supporting continued innovation. Future clinical development will also require standardized benchmarks for safety, immune reconstitution, long-term persistence, and therapeutic durability [1, 4, 237, 296].

### Failure modes and unintended immune consequences

Enhancing CAR-T cell activity does not necessarily translate into improved long-term therapeutic outcomes. Excessive CAR signaling, prolonged activation, sustained cytokine production, and excessive metabolic reprogramming may disrupt immune homeostasis, promote dysfunctional T-cell differentiation, and increase the risk of delayed toxicities. Accordingly, future CAR-T cell engineering should prioritize balanced functional optimization rather than maximal cellular activation, with designs tailored to the biological context of each disease.

As CAR-T cell engineering becomes increasingly sophisticated, the risk of unintended biological consequences also increases. Excessive CAR signaling, prolonged CAR-T cell persistence, and excessive metabolic reprogramming may promote T-cell exhaustion, impair lineage stability, and disrupt endogenous immune homeostasis. Consequently, prolonged CAR-T cell persistence may impair immune reconstitution and reduce responses to vaccination in the settings of autoimmune disease and chronic viral infection. Accordingly, optimization of CAR-T cell therapy should extend beyond maximizing efficacy and persistence to also preserve overall immune competence and physiological immune function [297, 298].

**Fig. 7:**
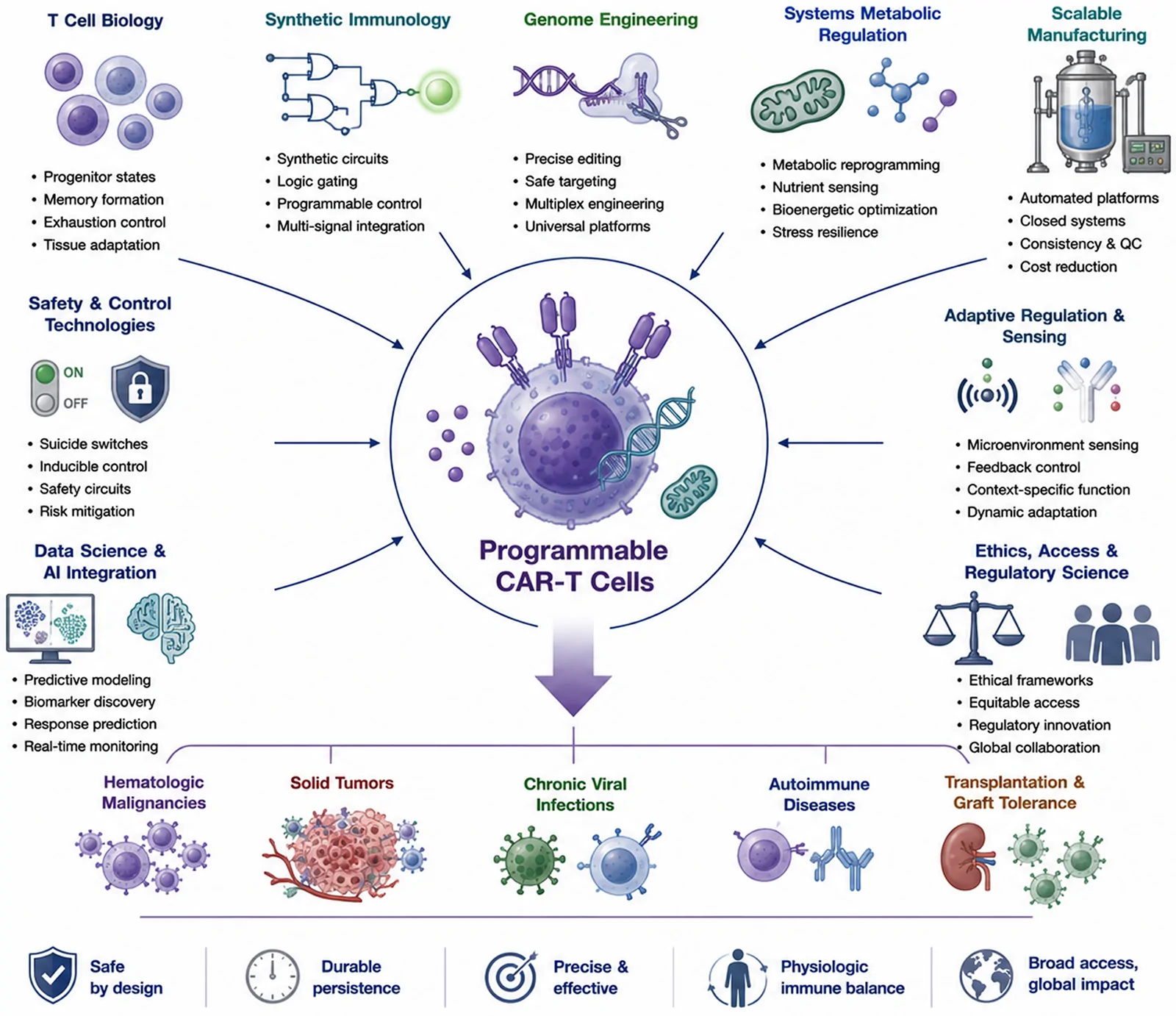
Future roadmap toward programmable cellular immunotherapy. The continued evolution of CAR-T cell therapy will depend on integrating advances in fundamental T-cell biology, synthetic immunology, genome engineering, systems-level metabolic regulation, scalable manufacturing, and adaptive regulatory frameworks. Collectively, these interconnected disciplines will enable the development of safe, durable, and clinically effective cell-based immunotherapies for cancer, chronic viral infections, autoimmune diseases, and transplantation while preserving long-term physiological immune function. *This conceptual framework was developed based on previous studies describing systems immunology*,* synthetic biology*,* genome engineering*,* programmable cellular therapies*,* and future directions in CAR-T cell therapy by Lim and June (2017)*,* June and Sadelain (2018)*,* Sadelain (2020)*,* Depil et al. (2020)*,* and Ho and Kaech (2022)*

Collectively, these considerations highlight that the future of CAR-T cell therapy will depend on achieving an optimal balance among efficacy, safety, manufacturability, and long-term immune regulation. Accordingly, the continued evolution of next-generation engineered cell therapies will depend not only on advances in CAR design but also on the integration of disease biology, synthetic immunology, and translational and clinical research (Fig. 7).

## Conclusions

Our understanding of CAR-T cell biology has evolved from viewing engineered T cells as static cytotoxic effectors to recognizing them as dynamic immune regulators whose function is shaped by antigen sensing, metabolic state, epigenetic regulation, and the tissue microenvironment. This conceptual shift has redirected CAR-T cell engineering beyond receptor optimization toward programmable regulation of cellular function across diverse disease contexts.

The findings discussed in this review suggest that future advances in CAR-T cell therapy will depend not on maximizing T-cell activation alone but on achieving an optimal balance among activation, persistence, resistance to chronic antigen stimulation, and adaptability to distinct immune environments. Advances in genome engineering, programmable gene regulation, and metabolic engineering are converging to enable safer, more predictable, and more effective cellular therapies.

Successful clinical translation of next-generation CAR-T cell therapies will depend not only on advanced synthetic gene circuits and multiplex genome engineering but also on controllability, manufacturability, long-term safety, and appropriate regulatory oversight.

Over the past decade, CAR-T cell therapy has evolved from a treatment for hematological malignancies into a versatile immunomodulatory platform with potential applications in solid tumors, chronic viral infections, autoimmune diseases, and transplantation.

Despite remarkable advances in CAR-T cell engineering, substantial challenges remain in scientific development, clinical translation, manufacturing, and regulatory implementation. The successful adoption of these technologically sophisticated cellular therapies will depend not only on continued scientific innovation but also on the demonstration of long-term safety, regulatory compliance, scalable manufacturing, and consistent efficacy across diverse patient populations. Consequently, future development should balance technological innovation with rigorous preclinical and clinical evaluation. This review has highlighted emerging biological concepts, including immune remodeling, long-term immune integration, and context-dependent regulation, that require further mechanistic investigation and clinical validation. Future clinical studies should incorporate longitudinal molecular profiling together with comprehensive functional immune analyses to better define the determinants of durable therapeutic responses.

Ultimately, the future of CAR-T cell therapy will depend not only on advances in CAR design but also on the integration of synthetic immunology, systems biology, manufacturing science, and clinical medicine into precisely regulated cellular therapies. Achieving an optimal balance among biological efficacy, physiological control, manufacturability, and clinical safety will determine whether CAR-T cell therapy can realize its full therapeutic potential across cancer and immune-mediated diseases.

## Data Availability

No datasets were generated or analysed during the current study.

## Abbreviations

APC: Antigen-presenting cell
CAR: Chimeric antigen receptor
CAR-T: Chimeric antigen receptor T cell
CAR-Treg: Chimeric antigen receptor regulatory T cell
CNS: Central nervous system
CRISPR: Clustered regularly interspaced short palindromic repeats
HBV: Hepatitis B virus
HIV: Human immunodeficiency virus
HSC: Hematopoietic stem cell
HSPC: Hematopoietic stem and progenitor cell
NK: Natural killer
PD-1: Programmed cell death protein 1
PD-L1: Programmed death ligand 1
sgRNA: Single guide RNA
SLE: Systemic lupus erythematosus
TME: Tumor microenvironment
Treg: Regulatory T cell
